# Enhancing Environmental Performance for Green Building Projects: Assessment Study due to the Applicability of Industry 4.0 Technologies

**DOI:** 10.1155/tswj/4976856

**Published:** 2026-07-16

**Authors:** Ismail W. R. Taifa, Omari Mperella

**Affiliations:** ^1^ Department of Mechanical and Industrial Engineering, College of Engineering and Technology, University of Dar es Salaam, Dar es Salaam, Tanzania, udsm.ac.tz

**Keywords:** applicable technologies, environmental performance, green buildings, Industry 4.0, Industry 4.0 technologies, sustainable development

## Abstract

The construction industry is embracing Industry 4.0 (I4.0) to improve environmental performance. This study thus evaluated the application of I4.0 on certified green buildings (GBs) projects. The study determined awareness level of I4.0 technologies on GBs, identified factors influencing I4.0 application, and proposed strategies for enhancing GBs′ environmental performance. The results of the awareness assessment showed that, among all I4.0 technologies, artificial intelligence (AI) was the most well‐understood. GBs apply some I4.0 technologies, including AI, Internet of Things, and cloud computing, and the awareness level for most I4.0‐related technologies was found to be high, ranging from 2.68 to 4.68 (for the scale of 1–5). The awareness level of the GBs′ principles was assessed, and the result showed that respondents were completely aware of two principles: energy efficiency and waste reduction. The overall mean score for the principles of GBs was 4.06, indicating a high level of awareness. The study also assessed the challenges of implementing I4.0, and noncritical challenges were a lack of standardization and certification, culture and aesthetics, client demands, and regulation and policy. The *R*‐value was 0.723, indicating a high correlation. The *R* ^2^ = 0.523 shows how much the predictor variables technology, top management commitment, staff skills, infrastructure, financial arrangement, and operational control factor can account for the variation in the environmental performance. Consequently, the study found perception‐based projections rather than outcomes verified through objective measurements that the I4.0 technologies are projected to enhance energy efficiency (15%–25%), water conservation (≥ 20%), improve indoor environmental quality, improve material selection and lead to waste reduction. Therefore, I4.0 technologies can enhance GBs′ performance by enabling real‐time monitoring and data‐driven optimization. I4.0 facilitates energy and resource management through smart systems, extends system lifecycles via predictive maintenance, and supports better environmental impact assessments in construction design. Despite challenges like high costs and cybersecurity risks, I4.0 technologies apply to both new and existing buildings, enabling dynamic, evidence‐based decision‐making that significantly improves environmental metrics and operational efficiency.

## 1. Introduction

The construction industry is increasingly drawing attention in many parts of the world to apply Industry 4.0 (I4.0) as a new technology. The installation of I4.0 captures the attention of construction activity as robotics and machines can operate in the construction activity [1, 2]. The application of I4.0 has greatly impacted the construction industry in terms of automation, which successfully indicates the rapid progress in the area of building methods, plants, materials, and machinery. The construction industry is planning to achieve the target through the application of I4.0 tools [3], but the construction sector, in most instances, is lagging behind modern industrial tools. The experience of the United Kingdom is improved through the “Made Smarter UK,” a review that identified construction as one of the sectors that could gain an advantage from the I4.0 revolution [4]. I4.0 tools need knowledge and information for more productivity while strengthening the efficiency that the construction sector demands. The construction industry′s pace is within digitalization through the advancement of data and connectivity of human machines. This is the robotics development to facilitate construction activity [5].

The twenty‐first‐century construction industry has been transformed in terms of the Internet of Things (IoT), increasing the reality of operation under the I4.0 perspective. The automation in advanced construction technology is designed to facilitate business operations [6]. The construction industry continues to advance into digitalization with substantial improvements in automation, productivity, and reliability, which are specific ingredients for green building (GB), whereas waste reduction and resource optimization achieve greater sustainability. That means the construction sector changes throughout the construction cycle, including planning, building, operation, and maintenance [7].

The construction industry has numerous projects, such as buildings, that are carried out with a great adverse environmental impact [8]. The building′s operation itself accounts for 40% of all energy usage [9]. It is one of the main causes of greenhouse gas emissions, responsible for 39% of global energy‐related emissions [10]. Despite this, the building construction activities that involve site clearance, excavation, and demolitions, as well as building operations that result in heating and cooling, water, and energy consumption, have become one of the leading sources of noise and air pollution to the environment and pose a great challenge to Architects, Engineers and construction stakeholders, hence becoming a threat to sustainability.

According to El‐kenawy et al. [11], the expanding global population necessitates the identification of sustainable and eco‐friendly alternative energy solutions, and the aforementioned strategy has resulted in a significant shift towards renewable energy sources. Some facilities, including buildings, use photovoltaic (PV) systems. The use of PV systems is currently prominent due to their decentralized energy production, scalability, and minimal environmental impact [11]. It is also crucial to recognize that smart city electricity forecasting is essential for the implementation of sustainable urban development, efficient energy distribution, and real‐time decision‐making to ensure infrastructure resilience [12].

GBs are promoted to create an environment for a green future [13]. GBs are relevant to reducing the adverse effects of building operations on the environment, occupants, and economy while maximizing the positive effects. The concept includes a range of principles such as energy efficiency, indoor air quality, resources, and water conservation [14]. Despite this, the buildings face challenges due to occupant behavior, lack of awareness and training, and long‐term maintenance. To overcome this, I4.0 is expected to revolutionize buildings through methods of operation and practice [15].

The I4.0 application is expected to facilitate the progression of GB [16]. In reality, less emphasis is placed on the pertinent application of I4.0 concerning the demand for higher efficiency and operation for GBs. Despite many advantages observed in other related industries regarding productivity, flexibility, efficiency, and sustainability, building construction, specifically GBs, lags in applying these I4.0 technologies due to a variety of factors required in GBs, which are more environmentally friendly than traditional buildings.

Moreover, numerous studies have been conducted on green construction, focusing on green design and GB materials [17]. Also, a few studies have examined I4.0 in facilitating GB [18]. However, little attempt has been made to improve the practice of I4.0 in GBs. I4.0 can aid GBs in operating effectively and efficiently, meeting the desired environmental and occupant‐related targets. Therefore, there is a need to assess the applicability of I4.0 technologies on GBs in Tanzania to enhance environmental performance. The main objective was to assess the applicability of I4.0 technologies to GBs to improve environmental performance in Tanzania. Three research questions achieved the main objective as follows: (a) What is the awareness level of I4.0 technologies on GBs? (b) What are the factors for applying I4.0 technologies to GBs to enhance environmental performance? and (c) What are the I4.0 strategies for GBs to enhance environmental performance?

## 2. Theoretical Orientation

### 2.1. I4.0 and GBs

#### 2.1.1. I4.0

I4.0 is recognized from the perspective of organizational and technological change, which comprises a large dimension of enterprises, institutions, organizations, factories, companies, and social changes related to technological advancement [19]. I4.0 is aligned with artificial intelligence (AI), the IoT, interconnectivity, machine learning, blockchain technology (BT), virtual reality (VR), and cloud computing (CC); these are applied based on cognitive computing, manufacturing, integration, smart manufacturing, dark manufacturing, big data analytics (BDAs), computer simulation, and relevant autonomous robotics [20, 21].

I4.0 is an advanced stage of industrialization, whereas the first stage was steam‐powered and focused on manual labor and simple tools utilizing traditional construction techniques like masonry, bricklaying, and carpentry [22, 23]. The second focuses on using prefabricated component construction techniques, standardized designs, and an early type of automation through an electrical system to boost productivity and expedite the construction process [24]. The third is centered on automation and computerization, utilizing digital project management, computer‐aided design, and advanced automation in construction to boost productivity, enhance design correctness, and foster improved teamwork and communication [25]. The fourth is focused on digitalization and smart technology, utilizing advanced digital twins, modular construction, data‐driven decision‐making, and autonomous robots to boost predictive maintenance, sustainability, and automated construction processes. The fourth industrial revolution is associated with technological advancement, which is digitalization [26, 27]. I4.0 applications intend to promote a green environment, based on the physical operations of the buildings in which organizations use machine learning and data processes to improve efficiency in processes and development.

#### 2.1.2. GBs

GB is a type of building constructed with relevant features to maintain or improve the quality of life based on the demands of the environment [28]. It is a structure and application with a specific process that creates efficiency in the building life cycle. It is ecologically sustainable and resource‐efficient over the whole building lifecycle, including planning, design, construction, operation, maintenance, renovation, and demolition [29]. The project relies on the tight collaboration of the contractor, architects, engineers, and clients throughout its phases [30]. GBs often enhance traditional design to optimize economy, usefulness, and comfort. It pertains to the conservation of resources via optimal energy efficiency, land utilization, water preservation, and material conservation [25]. The lifecycle of a building is synchronized with the environment to mitigate pollution and enhance health, comfort, and efficiency. GBs are fundamentally grounded on low consumption, high efficiency, environmental sustainability, economic viability, integration, and optimization.

#### 2.1.3. I4.0 Strategy in Construction

I4.0 technologies offer transformative potential for the construction industry, enabling more efficient, sustainable, and safe construction processes [31]. However, careful planning and training investment are essential to fully realizing its benefits. Table 1 shows the findings, strengths, and weaknesses of existing frameworks for the construction industry.

**Table 1:** Strategy strengths and weaknesses.

Source	Findings	Strength	Weakness
[32]	• I4.0 technologies have the potential to transform the construction industry.	• The strategy leverages IoT to connect the physical construction site with the cloud‐based cyber part, enabling real‐time data exchange and feedback loops for improved monitoring, decision‐making, and collaboration.	• Implementing this strategy requires significant upfront investment in technology infrastructure, software and training, which might not be feasible for all construction organizations.
	• CPS strategy is proposed to integrate these I4.0 technologies and improve construction capabilities.	• The strategy enables real‐time construction site monitoring, allowing for proactive decision‐making and adjustments based on current data.	• The strategy requires personnel with specialized skills in data analysis, CPSs, and CC.
			• Strategy focuses on the construction phase only and does not support other project phases, such as planning and design.
[25]	The strategy has a positive impact on environmental and economic sustainability.	The strategy can be applied to another context.	• The strategy focuses on the construction phase.
		• Inform policy development for GB.	• The strategy implementation is hindered by technology adoption due to cultural and personnel skills.

### 2.2. GB Theory

The GB theory was developed in 1990 by Pei in the UK; this was advanced at the United Nations Conference towards the idea of advancement in the standard and required direction aligned with Green development [29]. The building theory intends to balance creating structures and promoting green development. The theory is relevant in buildings with lower maintenance costs and engineered elements with specific lower energy consumption, which ultimately reduce energy costs and water bills [8]. The theory failed to articulate the high initial investment, accessibility of the right materials, long time to build, difficulty controlling indoor air temperature, selecting the right location, and finding the right laborers [33]. The theory of GB is significant because it emphasizes the construction of structures that promote green development. This is relevant to this study because it focused on applying I4.0 to GBs.

### 2.3. Empirical Review

#### 2.3.1. Awareness Level of I4.0 on GBs

Müller et al. [34] studied the drivers of the I4.0 application. Implementation of I4.0 is related to the awareness level on the impact of I4.0 value creation based on the opportunities and challenges, and the practical and theoretical concerns on the application of I4.0, and the awareness level encourages dynamics of implementation. The I4.0 pertinent challenges and opportunities are conceptualized in terms of size, sectors, and the role of I4.0 providers. Findings noted that dimensions of strategy, operation, environmental, and social opportunity are drivers for awareness level based on the respective implementation [34]. The challenges with regard to awareness, implementation, viability, and sustainability tend to impede the progression of GB and its effectiveness through the application of I4.0 in the sustainability context.

Safar et al. [35] explored the awareness of I4.0 on sustainability in India. Approaches, strategies, and technologies related to I4.0 are becoming more important to obtain competitive advantages for GB. Unlike earlier research, which primarily focuses on sustainability‐specific attempts based on the widespread awareness of I4.0 concepts in the South Indian region, by conducting a questionnaire‐based survey on residents. The results showed a very low degree of knowledge about the I4.0 idea and its constituent parts, which subsequently result in insufficient expectations and future actions towards GBs [35]. Furthermore, those who have previously learned about the I4.0 strategy tend to have optimistic views and expectations for potential future developments. The lack of I4.0 awareness is a primary obstacle to a prosperous and long‐lasting transition in the I4.0 revolution [35].

Ersoz et al. [36] studied I4.0 awareness in Turkey. Technology is developing at a rapid pace, especially in the area of AI, which has fueled the idea of I4.0. The study found that the nature of the organization significantly impacts I4.0 awareness levels. It is critical to draw attention to any problems that an organization encounters in a transition stage from the obsolete industrial idea to the more contemporary I4.0 idea [36]. Therefore, this study aims to evaluate the level of I4.0 awareness among Turkish organizations. The study also seeks to determine how certain I4.0 laws and training programs vary in relation to key demographic characteristics of Turkish organizations. Findings demonstrated that educational backgrounds influenced awareness of I4.0. Ersoz et al. [36] also discovered that the kind or extent of these organization relationships had a major influence on I4.0 awareness levels.

Tiep et al. [37] conducted a study on the degree of knowledge and the variables influencing the GB trend in Pulau Pinang, Nigeria. Since these organizations are the primary industry influencers, the participating enterprises have a background in development. Since property developers are the first parties to start a construction project, they are essential in promoting GB techniques.

##### 2.3.1.1. Design, Methodology, and Approach

To determine the awareness level and the factors influencing the awareness level, thorough literature research is first carried out. The study has since adopted a qualitative methodology, gathering insights from industry experts to confirm the authenticity of information gathered via the literature review [37].

##### 2.3.1.2. Findings

Based on the data gathered, it can be concluded that there is still room for improvement in the Penang construction industry′s awareness level [37]. Although most developers recognize the value of green practices, they do not implement them for a variety of reasons, including lack of funding, insufficient experience, and insufficient stakeholder intention in introducing green practices into projects.

##### 2.3.1.3. Limitations and Consequences of the Research

The primary goal of this work is to determine the degree of awareness regarding the Penang building sector. Influential developers in the Penang building sector provide information about factors that impact awareness levels; however, not all developers choose to participate in the study.

Balasubramanian et al. [25] addressed the level of awareness of stakeholders of GBs in Indonesia, saying that GBs took off immediately and became popular all over the world. Approximately 40% of all CO_2_ emissions and more than a third of global energy consumption are attributable to the building and construction sector [37]. It is well‐known that GB practices reduce CO_2_ emissions and energy consumption. It is necessary to understand the importance of implementing the GB concept for its successful deployment. The goal of this study is to examine the degree of knowledge among construction stakeholders (i.e., how well they comprehend GB and its rules, how significant they perceive GB to be), as well as how they come to understand the notion of GB [37]. Descriptive statistics were used to examine the questionnaires that were given to different stakeholders. The survey indicates that Indonesian individuals employed in construction settings are knowledgeable about and cognizant of the idea of GB. Nonetheless, many remain uninformed about legislation and consider government assistance essential. Thus far, they have acquired knowledge about GBs from lectures and media sources. Additional study is required to ascertain the most efficient technique for promoting green construction. The findings indicate that Indonesian individuals employed in construction settings had knowledge and awareness of the GB concept. Nonetheless, many remain uninformed about legislation and consider governmental assistance essential [37]. Until now, they have gained an understanding of GBs through lectures and the media.

Sajjad et al. [22] studied the construction industry′s evaluation of GB awareness. The financial and environmental elements influencing the adoption of GB in Jordan and ranking them according to significance, this study seeks to give a clear picture of the degree of understanding of GB principles among Jordanian construction enterprises. Additionally, obstacles to green initiatives in Jordan were noted. A well‐designed questionnaire was randomly given to 210 clients, consultants, and contractors. Seventy‐six completed surveys were sent back. Measuring quantitative data, it was found that only 32% of respondents had experience working on a GB project. The majority concurred that lower operating and maintenance costs were the most motivating aspects of GB implementation, whereas the main obstacles to the successful completion of green projects were perceived to be additional costs and time, a lack of government support, and a lack of green expertise [22].

Recommendations were made to reduce the impact of the barriers in light of these findings [22]. This study also provided guidelines for successfully managing GB projects. An experienced project manager, extra effort in managing budget and schedule, a high‐standard communication system, and stakeholder involvement in all stages were aspects that respondents strongly felt were required to obtain desired results. Many people are concerned about a new project′s economic sustainability, especially developers involved in the building process. Thus, the study′s focus was on financial variables that could have an impact on Jordan′s GB implementation. One of the aspects that respondents agreed on the most was cheaper operating and maintenance costs. Two times as much for design and construction.

According to the findings of research conducted by Anzagira et al. [38] on the knowledge and implementation of green building codes (GBCs) in Sub‐Saharan Africa, the construction sector is experiencing environmental issues, which have resulted in a slower acceptance rate when compared with industrialized countries. According to the results of a questionnaire study that was administered to 292 stakeholders, 88.4% of respondents had previous awareness of GBCs, and 69.2% of respondents said that they are environmentally sustainable [38]. Energy efficiency and interior environmental quality had the greatest awareness ratings, with 97.6% and 93.8%, respectively, saying that they were the most important [38]. GBCs are used to a considerable degree in Ghana, with One Airport Square in Accra being the most extensively utilized venue. According to the findings of the research, the internet is the most effective medium for disseminating information about GB, which might possibly promote the adoption of the Ghanaian Construction Industry (GCI) GB. Table 2 depicts the awareness level of I4.0 on GBs.

**Table 2:** Awareness level of I4.0 on GBs.

Awareness level	Nature of the project	Model or method	Source
High aware	Private building	Questionnaire	[34]
Very low degree of knowledge	Government project	Survey questionnaire	[35]
High awareness	Government project	Questionnaire	[36]
Low awareness	Government project	Interview	
High awareness	Government project	Questionnaire	[25]
High informed	Government project	Questionnaire and interview	[19]
High awareness	Government project	Questionnaire	[22]
Low awareness	Government project	Questionnaire	[22]

#### 2.3.2. Factors for I4.0 on GBs

Although I4.0 has the potential to support GB development, building design has not yet given most of these themes significant thought [39]. The goal of I4.0 is to achieve GB. In order to prolong service life and minimize negative effects on the economy and environment, extremely GBs are required for the constant reconfiguration and extension of I4.0. However, the majority of studies and instruments concentrate on promoting GB. In order to meet the demands of I4.0, this study offers a specific guideline for adaptable industrial structures that integrate building planning with I4.0. The study found several factors that could be used to integrate I4.0 into GB: flexibility, planning, data‐driven decision‐making, early design stage, and resource efficiency, including energy optimization, water conservation and material optimization.

Sajjad et al. [22] assessed the I4.0 in the construction industry. I4.0 has emerged during the past 5 years as a disruptive force with a wide range of effects on the construction sector. Implementing I4.0 technology is one of the most important factors. Their study implemented a multicriteria decision‐making methodology to assess construction companies′ strategic preparedness for implementing I4.0. Various models have been developed in the literature to assess the level of development towards I4.0. However, no paradigm for assessing strategic preparedness has yet been developed. Furthermore, none of them addressed the building sector. The findings showed that the factors for implementing I4.0 in GB are human capital, including construction experts, intellectual agility, knowledge, abilities, and competencies [22].

Bachurinskaya et al. [40] examined the variables influencing innovation in the construction sector. The research seeks to identify the elements influencing the creative advancement of the building and investment sector within I4.0 processes. The objective is to develop digitalization models for construction and investment firms informed by the dissemination of I4.0 trends in Russia [40]. The investment and construction sectors of GBs within the socioeconomic framework are influenced by several variables. The determinants for the implementation of I4.0 in GB include procedures, investment and construction, digitalization, ecology, innovation, and location [40].

Governments globally are addressing the sustainability challenges confronting the construction sector, which include elevated carbon emissions, health and safety hazards, diminished productivity, and escalating costs [25]. Certain challenges may be addressed by the use of I4.0 technology in the construction industry, often referred to as Construction 4.0 (C4.0). Nonetheless, our current comprehension of this matter is significantly restricted, since previous studies have been notably disjointed and limited to the technologies used and their connection to the triple bottom line of sustainability perspectives. Balasubramanian et al. [25] determined that, although the repercussions are analogous for social sustainability, the advantages of C4.0 on environmental and economic sustainability surpass its disadvantages.

The implementation of C4.0 technologies was shown to be inconsistent; building information modeling (BIM) and automation exhibited a more significant utilization compared with other technologies, such as cyber‐physical systems (CPSs) and smart materials. Blockchain and three‐dimensional (3D) printing technologies are anticipated to see substantial expansion in the future. The proposed creative technique may facilitate the establishment of support systems and policy interventions to enhance the deployment of C4.0 while alleviating its adverse impacts on sustainability. It is also feasible to modify and implement the approach in various national and industrial contexts.

Sajjad et al. [22] evaluated the success of I4.0 digitalization practices. The construction industry has closely monitored the potential of I4.0 digitalization processes to enhance overall project performance and promote sustainability. Consequently, empirical research examining the particular components and ideas that contribute to its achievement is essential. By investigating the positive effects of I4.0 digitalization approaches in sustainable building management, this study seeks to close the gap in the literature. Survey data from China′s construction industry were examined using a mixed‐methods approach that combined structural equation modeling (SEM) and exploratory factor analysis (EFA) [22]. Concepts related to technology, design, sustainability, functional elements, resource management, and managerial effectiveness were included in the survey questionnaire. The idea of sustainability has been found to have the greatest influence on developing sustainable building techniques [22]. This study contributes to the growing body of knowledge about what makes I4.0 digitization methods within sustainable construction management successful.

Newman et al. [30] concentrated on the implementation of I4.0 within the construction sector. I4.0 is anticipated to revolutionize industrial and commercial processes by enhancing efficiency and the use of information. Nevertheless, other challenges persist, and the construction industry remains notorious for its sluggish adoption of innovations. The outcomes of the literature study indicate that research on I4.0 remains nascent, with industrialized countries, particularly Germany, at the forefront [30]. Although a plethora of alternatives exists, there are also some impediments to enabling [30]. The review′s conclusions are substantiated by the case study′s findings, which indicate that although practitioners are optimistic about I4.0′s potential to rejuvenate the construction sector, its implementation is nonetheless obstructed by persistent managerial practices that depend on human interaction. Building modeling information (BMI) is now garnering significant attention from industry professionals, sometimes at the expense of other, more advanced I4.0 technologies [30]. Table 3 depicts some of the factors for I4.0 on GBs.

**Table 3:** Factors for I4.0 on GBs.

Factors	Source	Nature of the project	Model or method
Presence of skilled manpower.	[41]	Government buildings	Questionnaire
Flexibility, planning, data‐driven decision‐making, early design stage, and resource efficiency, which include energy optimization, water conservation, and material optimization.	[39]		
Practitioner acceptability, construction environment, attitude and inductive reasoning were carried out.	[30]		
Human capital includes experts in the construction process, intellectual agility, knowledge, abilities, and competencies.	[22]		
Policy intervention	[25]		
The revealed interconnected constructs provide valuable insights for achieving sustainability, enhancing technology adoption, optimizing design processes, streamlining functional elements, improving resource utilization, and increasing managerial efficiency.	[42]		Questionnaire and interview
Processes, investment and construction, digitalization, ecology, innovation, and location.	[40]		

#### 2.3.3. I4.0 Strategies for GBs

I4.0 strategy positively impacts environmental and economic sustainability [25]. The strategy can be applied to another context. Inform policy development for green construction. The strategy focuses on the construction phase. The implementation of the strategy is hindered by technology adoption due to cultural and personnel skills [25].

Chen et al. [19] argued that, as it encompasses the idea of sustainable development, the smart city now provides the ideal condition of urban growth. BIM is becoming a vital tool for the construction industry, and C4.0, which has its roots in I4.0, is just two of the cutting‐edge ideas and technologies being integrated into the construction industry to create a smart city. Thus, in the smart city context, Chen et al. [19] seek to examine the state of the art and future directions of the multidisciplinary integration of C4.0, I4.0, BIM, and I4.0 technologies, which have the potential to transform the construction industry [32]. A CPSs strategy is proposed to integrate these I4.0 technologies and improve construction capabilities. The strategy leverages IoT to connect the physical construction site with the cloud‐based cyber part, enabling real‐time data exchange and feedback loops for improved monitoring, decision‐making, and collaboration. The strategy enables real‐time construction site monitoring, allowing for proactive decision‐making and adjustments based on current data [19]. Implementing this strategy requires significant upfront investment in technology infrastructure, software, and training, which might not be feasible for all construction organizations. Some studies that proposed strategies are in Table 4.

**Table 4:** I4.0 strategies for GBs.

I4.0 strategies	Nature of the project	Model or method	Source
Systematic strategies for advancing sustainable building development	Government project	Questionnaire and interview	[19]
Policy strategy			[25]
Enabling strategies		Questionnaire	[25]
GB strategies			[43]
Cyber‐physical system strategy			[32]

#### 2.3.4. GBs and Sustainability

Patel and Patel [29] applied GB materials in the construction improvement towards green development. This is aligned with the benefits, sustainability, and respective emphasis in the construction activity. Sustainability involves three tenets: environmental, economic, and social factors [44, 45]. Therefore, GBs have a direct relationship with environmental performance because the environmental aspect is a subset of sustainability [46–48]. Sustainability is increasingly drawing attention in terms of scale in GBs, which is more relevant in the construction industry [29]. The environmental challenges are increasing as a result of development, particularly urbanization. These need to be integrated into the construction industry by applying GB materials. These tend to reduce pollution and environmental problems while improving the environment. This is expected to be achieved by applying recycled design materials in the GB. This has increased the demand for GBs as materials are environmentally friendly. They mainly protect both the economy and the environment, and the energy supply is used in a way that is friendly to the environment [29].

Rahmayanti et al. [8] focused on the role of GB in I4.0. The building sector significantly impacts the community throughout the transition cycle and is a major outcome of the construction sector, generally reflecting this. Both constructive and destructive criticism exist. Buildings and construction‐related negative effects interfere with everyday human activities like talking, discussing, traffic congestion, air pollution, and garbage disposal. Following completion, the building blends into its surroundings. To overcome the challenges of developing these structures, adequate technical innovation support is required [8].

Hager et al. [49] concentrated on I4.0 and sustainability. The discourse around I4.0 and sustainability has gained momentum in academic, managerial, and policy domains. Notwithstanding the significance of the topics, the relationship between I4.0 and sustainability, as proposed by several writers, remains ambiguous; the literature is fragmented. This study seeks to circumvent this limitation by conducting a comprehensive literature review of 117 peer‐reviewed journal articles. Hager et al. [49] give a conceptualization and theoretical framework following descriptive and content analyses. The study advances knowledge of I4.0 and sustainability, particularly the effects of I4.0 technology on sustainability practices and performance. This advances both theory and practice.

Javaid [1] focuses on understanding how I4.0 technologies are being adopted to enhance sustainability in the environment. This study examines how I4.0 innovations support the development of green environments in manufacturing and other sectors [1]. I4.0 technology and the vital connections that these cutting‐edge technologies establish should be beneficial to the environment. Manufacturing is closely connected to information and communication systems in the era of I4.0, which increases its scalability, competitiveness, and knowledge. I4.0 provides a range of guidelines and technologies for developing new and current factories with scalable robotics, information and communications technology [1, 50]. This enables clients to choose from a range of models at production rates.

### 2.4. Research Gap

The present literature failed to articulate I4.0 in GB, specifically on the implications of applying I4.0 technologies for enhancing environmental performance. The findings from the reviewed literature failed to clearly articulate the way I4.0 could be integrated into GBs, where the adoption process had not yet been fully implemented. Some studies on GBs mainly focus on the effect on the environment or the sustainability concepts in general [48]. Other studies detail the sustainability considerations of GBs without emphasizing the need for I4.0 [51, 52]. Consider this specific concern from the reviewed literature on green materials in the construction of GBs [29]. There are limited studies on the relationship between GBs and sustainability. Examples of such studies include Javaid [1], who strongly focused on comprehending how I4.0 technologies are being adopted to improve environmental sustainability. Likewise, Beltrami et al. [53] focused on I4.0 and sustainability. The reviewed literature indicated that few attempts were made to apply I4.0 to GBs, where Tanzania could be appropriate for studying this. Therefore, there is a need for a study to address the need for assessing the applicability of I4.0 by considering the GBs mainly on environmental performance.

### 2.5. Conceptual Framework

**Figure 1:**
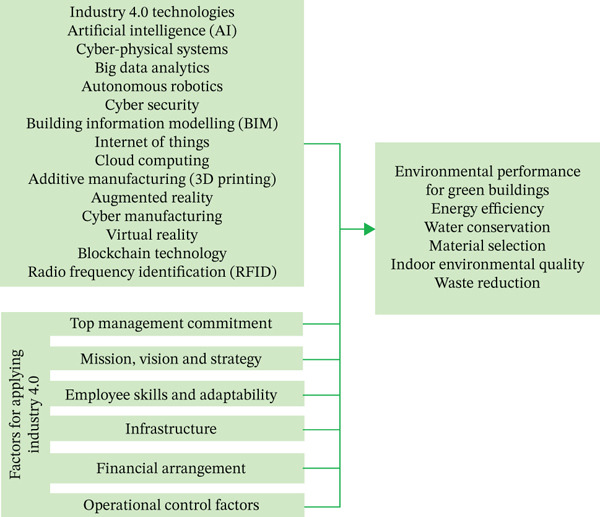
Conceptual framework.

The independent variables include the awareness level of various I4.0 technologies (e.g., AI, integration, factors for application, and strategies). Such an independent variable influences the dependent variable, that is, environmental performance in GBs. Some indicators of environmental performance measurement include indoor environmental quality, water conservation, energy efficiency, material selection, and waste reduction for GBs. Figure 1 shows the conceptual framework for exploring I4.0 on GBs.

Regarding Figure 1, the factors for applying I4.0 to GBs to enhance environmental performance are as follows. The technology involved ranking sub‐technologies such as AI, CPSs, BDAs, autonomous robotics, cybersecurity, simulation, the industrial IoT, CC, additive manufacturing (3D printing), augmented reality (AR), cyber manufacturing, VR, and blockchain technology (BT). Top management commitment involved subfactors in encouraging the use of I4.0, assessing knowledge on the use of I4.0 technologies, and ranking the support and engagement in utilizing I4.0 technologies. Mission or vision and strategy involved assessing the presence of projects′ vision and strategy aligned with the I4.0 concept and the plans or processes for implementing I4.0 in enhancing environmental performance. Employee skills and adaptability factors were checked based on knowledge of I4.0 technologies, skills in applying I4.0 technologies, competence to use I4.0 technologies, and whether the GB project recognized the benefits of implementing I4.0 technologies. The infrastructure factor looked at available ICT systems and the reliable internet to support I4.0. Financial arrangements involved the availability of financial resources, budget allocation, financial software to manage GBs, and an I4.0‐specific budget. Operational control factors involved looking at the applicability of I4.0 for enhancing environmental sustainability, I4.0 technologies deployment and integrating I4.0 technologies to enhance environmental sustainability in the GB.

## 3. Research Methodology

### 3.1. Research Design

This study used a cross‐sectional design because it facilitated data collection at a single point in time from respondents of different characteristics. Within such a design, a mixed‐method approach was used because it combines quantitative and qualitative approaches. It allowed data collection in terms of numerals and descriptions that enhanced the level of I4.0 on GBs in Tanzania.

The research focused on completed GB projects in Tanzania that are certified by the US Green Building Council (USGBC). Further, the building construction industry is often categorized into three construction cycle stages, including planning, building, operation, and maintenance. The emphasis is on assessing the applicability of I4.0 technologies on GBs to enhance environmental performance in Tanzania.

The population is a list of items applied for an investigation that serves as the study′s sample. It includes various elements such as people, companies, and production units with common characteristics. The population for the study included expert respondents, such as engineers, quantity surveyors, and architects, who participated in the building’s design, construction, and operation. Those experts were 123 among those who participated fully in constructing the Evoke Luminary building, Kambarage House (National Housing Cooperation Building), Kigamboni Housing Estate, and the Citibank building projects.

The unit of analysis is the major entity used to analyze the particular situation in the respective study. In order to assess the I4.0 technologies on GBs in Tanzania, the sampling unit consisted of individuals, quantity surveyors, architects, and engineers. Sampling is essential in any research activity. It is theoretically difficult to analyze the entire population due to time and financial constraints. The study is aimed at getting adequate information on GB projects in Tanzania. So, this research focused only on certified projects. Based on these criteria, seven buildings were completed and certified as GBs in Dar es Salaam, Mwanza, and Zanzibar. However, the research only focused on four GBs in Dar es Salaam. The study used a purposive sampling technique to select respondents based on their professional background, education and experience, knowledge of sustainability and GB projects, among others. The participants on the four GB projects were as follows: Evoke Luminary building (28 participants), Kambarage House (National Housing Cooperation Building) (28 participants), Kigamboni Housing Estate (33 participants) and Citibank building (34 participants). The respondents from each GB involved quantity surveyors, architects, clients, site engineers, technicians, facility managers, consultants, site managers, environmental expert and contractor representatives among the four GBs in Dar es Salaam (Table 5).

**Table 5:** Sample size.

Participants	Evoke luminary building	Kambarage house	Kigamboni housing estate	Citibank building	Total
Quantity surveyors	1	1	1	1	4
Architects	1	1	1	1	4
Clients	1	1	1	1	4
Site engineers	11	10	15	14	50
Technicians	6	7	7	9	29
Facility manager	1	1	1	1	4
Consultant	1	1	1	1	4
Site managers	1	1	1	1	4
Environmental expert	1	1	1	1	4
Contractor representatives	4	4	4	4	16
**Total**	28	28	33	34	123

### 3.2. Data Collection Methods and Techniques

The study collected data through questionnaires, an observational approach, and a documentary review.

#### 3.2.1. Questionnaires

A questionnaire is an instrument that is applied with a series of questions to facilitate data collection [54]. The study used a closed‐ended questionnaire (File S1), which was administered to all 123 participants, as shown in Table 5. The pilot test was performed to check the validity of the questionnaire. This study conducted face validity and pilot testing by sending the questionnaire to seven respondents; only those who were not part of the main respondents were considered. The seven participants for the validity testing included two engineers, one architect, two quantity surveyors, and two clients who were progressing well in accrediting one GB in Dar es Salaam. Their responses were descriptively analyzed, and all suggested changes were incorporated. Testing the reliability of the questionnaire also involved the determination of the composite reliability (CR), which represents the internal consistency of the construct; thus, concluding whether the measured applicability of I4.0 technologies for GBs. Likewise, the study computed the average variance extracted (AVE).

#### 3.2.2. Observational Approach

Observation is specifically aligned with collecting data through watching events, behavior, or physical characteristics in the natural environment. Certified GBs were visited to observe and see how the application of I4.0 is carried out, as well as the operation of buildings in terms of energy efficiency, water conservation, material selection, indoor environmental quality, and waste reduction.

#### 3.2.3. Document Review

Documentary review involves information that has already been acquired by someone else and has undergone statistical analysis. Documentary review in this study included reviewing building project reports, building laws, and regulations. The documents were accessed from certified boards. The documents were for the four buildings: Evoke Luminary building, Kambarage House (National Housing Cooperation Building), Kigamboni Housing Estate, and Citibank building. The reports were up to those recorded by December 2024. In some documents, advanced technologies were used for some buildings, while other qualitative details were also extracted, including the features of the buildings.

### 3.3. Data Analysis Techniques

Quantitative data were analyzed using the Statistical Package for the Social Sciences (SPSS) Version 21.0. Data analysis methods were presented for each objective. The validity of the questionnaire was determined as follows: before sending out 32 questionnaires to experts, a pilot study was conducted to ensure that the study collected the necessary data. Eight questionnaires were distributed to respondents, and their responses were examined to see if they were useful for the study. The degree to which research findings are valid over time and fairly reflect the population being studied is known as reliability [55]. To guarantee test validity, internal consistency must be established prior to using a test for research or assessment [56]. Thus, the validity of the collected data was reached using Cronbach′s alpha in SPSS 21.0. According to [56], the minimum value of Cronbach′s alpha for internal consistency should be at least 0.7 [57]. The study also performed convergent and discriminant validity. This was performed to make sure that a measurement tool accurately measures the desired concept. Convergent validity shows that measures of the same construct are quite similar, whereas discriminant validity shows that measures of separate constructs are not or have weak relationships.

**Figure 2:**
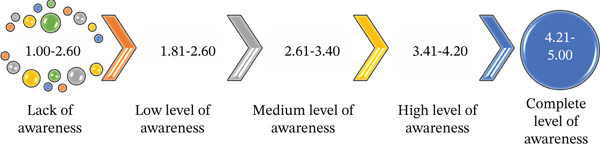
Ranges for 5‐point scale options for items. Source: Eski et al. [58].

For the first objective, a descriptive analysis of the awareness level of I4.0 on GB was used. The descriptive analyses included mean, standard deviation, and relative importance index (RII). The awareness level was assessed based on Eski et al.′s [58] study (see Figure 2). Despite that establishing the “awareness level” based on the scale (1–5) may be found arbitrary for other studies, the awareness level for this study considered Eski et al.′s [58] study to validate its application.

The second objective was to use the factors for I4.0 on GB descriptive statistics of absolute and relative (percentages) and frequencies. For the extraction of the factors, the components were extracted using EFA in SPSS 21.0. The EFA was performed using factor loadings of more than 0.5 for each factor on its variables, and components were recovered using eigenvalues greater than one [59]. EFA used the principal component analysis (PCA) through varimax rotation. In the analysis, six components were extracted using PCA. Therefore, rotation was both applicable and performed to improve the interpretability of the factor structure.

Also, the study explored factors for applying I4.0 technologies to influence environmental performance. This was done by conducting linear regression analysis in SPSS 21.0. A linearity test was conducted, followed by a regression analysis. To conduct linear regression analysis, the linear relationship between dependent and independent variables is initially assumed [60]. As such, the relationship that exists between the predictor (independent) variable and the outcome (dependent) variable can be modeled and represented as a straight line. This reasoning is fundamental owing to the fact that linear regression models attempt to identify the line that best fits the relationship between the two variables, which, if found accurately, will yield dependable inferences, unlike the case where there are nonlinear relationships within the data. The linearity test was conducted to ensure the linear relationship between the variables. The linearity test aimed to reveal a linear relationship between all independent variables through the significance values of the deviation from linearity. The independent variable is considered linearly related to a dependent variable if it has significant values for the deviation from the mean greater than 0.05 [60]. Their results were summarized via the analysis of variance (ANOVA). The regression model is shown by Equation (1).

(1) Y=β0+β1X1+β2X2+β3X3+β4X4+β5X5+β5X5+β6X6+Ɛ,

where *Y* is the environmental performance, *X*_1_ to *X*_6_ represent technology, top management commitment, employee skills, infrastructure, financial arrangement, and operational control factors; and *Ɛ* represents the error term.

Also, an assessment of I4.0 technologies was conducted. The gathered responses were descriptively analyzed using the mean scores and RII to determine which technologies were more crucial to use in GBs than others. To analyze and prioritize the challenges associated with the adoption of I4.0 technologies in GBs, this study employs the multidimensional normalization approach proposed by Mukhametzyanov [61]. This method is particularly suitable for handling heterogeneous data derived from Likert‐scale responses, where variables may differ in distribution and measurement scales. Given that the problem involves evaluating multiple interrelated technical, financial, and institutional factors, it aligns with a multicriteria decision‐making framework. The normalization process ensures scale conformity, reduces bias associated with subjective responses, and enables the computation of normalized mean values used to rank the relative importance of identified challenges.

Compared with conventional techniques such as mean score ranking and RII, this approach provides greater mathematical rigor and comparability across multidimensional data. Although qualitative frameworks like SWOT (strengths, weaknesses, opportunities, and threats) analysis are useful for categorizing challenges, they do not support quantitative prioritization. The adopted method therefore complements such frameworks by enabling systematic ranking of challenges based on their relative significance. Its application is particularly relevant in the Tanzanian context, where data are often perception‐based and lack standardization, making it a robust and practical tool for supporting evidence‐based decision‐making in the adoption of I4.0 technologies in GBs.

### 3.4. Stages for Developing the Strategies

The applied research strategy is categorized into two main stages. The first is the literature review stage of developing the application of I4.0 on GBs. The gathered response involved EFA. EFA was conducted in SPSS 21.0 for the strategies to guarantee the alignment of all items assessing strategies in a single variable.

### 3.5. Ethical Considerations

The study collected data through questionnaires, an observational approach, and a documentary review. Thus, all ethical issues include informed consent, voluntary participation, confidentiality and anonymity, respect for participants, and transparency and accountability. We also provided the consent form before collecting data from participants. Only participants who read and understood the informed consent sent or provided to them were engaged in the study. Therefore, quantity surveyors, architects, clients, and engineers were all involved voluntarily.

## 4. Results and Discussion

The study′s results were examined and presented in this section in accordance with each particular objective. The study is aimed at evaluating how I4.0 technology could be used to increase Tanzania′s GB sustainability. The study′s first specific objective was to ascertain the extent of awareness regarding I4.0 technologies in GBs. Subsequently, it sought to identify the factors that should be considered when implementing these technologies in GBs. Finally, the study suggested various strategies for implementing these technologies in GBs.

### 4.1. Validity, Reliability Tests, and Sampling Adequacy

Validity determines true research results by assessing whether the study measures what it was designed to test. It is carried out by posing a sequence of queries and contrasting the answers with those of other studies [55]. Before sending out the mass questionnaires, a pilot study was conducted to make sure the study collected the necessary data. Eight questionnaires were distributed to respondents, and their responses were examined to see if they were useful for carrying out the study. As a result of the sample study, some questions were changed to ensure consistency and simplicity with the research.

The degree to which research findings are valid over time and fairly reflect the population being studied is known as reliability [55]. To guarantee test validity, internal consistency must be established prior to using a test for research or assessment [56]. Using Cronbach′s alpha, a measure of the internal consistency of the responses provided to the questionnaires, the reliability test was carried out in SPSS 21.0. According to Table 6, the aggregate Cronbach′s alpha for the data collected was 0.985, whereas for each study variable, the alpha was above 0.917. According to Saoula et al. [56] and Twaha and Taifa [62], both satisfy the necessary minimum values of Cronbach′s alpha for internal consistency, which is 0.7.

**Table 6:** Reliability test results.

Items	Cronbach′s alpha	Cronbach′s alpha based on standardized items	*N*of items	Composite reliability	Average extracted value (AVE)
Awareness	0.966	0.951	31	0.745	0.759
Principles of GB	0.957	0.960	5	0.942	0.933
Environmental performance	0.959	0.948	10	0.780	0.635
Challenges	0.913	0.917	10	0.939	0.906
Factors	0.952	0.952	33	0.734	0.912
Strategies	0.958	0.959	13	0.867	0.659
Overall	0.985	0.985	115	0.890	0.884

The study performed CR, which represents the internal consistency of the construct; thus, concluding whether the measured applicability of I4.0 technologies for GBs. For the acceptable CR, the threshold value should be 0.70 [63]. The study′s results in Table 6 show that the CR values are at least 0.7, thus implying that the tool for collecting data was valid. Likewise, the study computed the AVE: AVE measures the amount of variance captured by the construct in relation to the variance due to measurement error, and the threshold is typically > 0.50. The AVE values in Table 6 prove that all measured items were at least 0.50, hence validating the use of the data collection tool. All found that all items′ standardized factor loadings and AVE were over 0.5, which means that they all had good convergent validity.

Kaiser–Meyer–Olkin (KMO) was performed to measure the sampling adequacy. Also, the study performed Bartlett′s test of sphericity and established communalities for all items using SPSS 21.0 software before performing EFA. The inclusion of KMO and Bartlett′s test is essential in EFA as they assess the suitability of the dataset for factor extraction. The KMO statistic evaluates the proportion of variance among variables that may be common variance, with values above 0.6 indicating that the sample is adequate for reliable factor analysis. In this study, the reported KMO value exceeds the recommended threshold, confirming that the correlations among variables are sufficiently compact to produce distinct and reliable factors. Bartlett′s test of sphericity, on the other hand, examines whether the correlation matrix significantly differs from an identity matrix. A statistically significant result (*p* < 0.05) indicates that meaningful relationships exist among variables, justifying the application of factor analysis. The results in this study meet this criterion, supporting the appropriateness of EFA.

Yusoff et al. [64] state that to demonstrate discriminant validity, the square root of the average value extracted (bold diagonal values) for each construct must be larger than any other construct. Likewise, the cross‐loading items′ value must be greater than any other construct′s. The variables have strong discriminant validity, as indicated by the explanation of the criteria being met. All found that all items′ standardized factor loadings and AVE were over 0.5, which means that they all had good convergent validity. From Table 7, the discriminant validity for the items tested was all above 0.5.

**Table 7:** Discriminant validity for the items tested in the questionnaire.

Tested items	AWW	PRI	ENV	CHA	FAC	STR
Awareness (AWW)	**0.871**					
Principles of GB (PRI)	0.347	**0.966**				
Environmental performance (ENV)	0.773	0.351	**0.797**			
Challenges (CHA)	0.445	0.627	0.127	**0.952**		
Factors (FAC)	0.328	0.475	0.136	0.305	**0.955**	
Strategies (STR)	0.354	0.021	0.081	0.214	0.593	**0.812**

*Note:* The bolded diagonal values are all greater than the corresponding off‐diagonal correlation coefficients in their respective rows and columns. This confirms that each latent construct has stronger relationships with its own indicators than with other constructs.

### 4.2. Demographics of Respondents

The total number of respondents was 123. The demographic characteristics and information of respondents are summarized in this section. The demographics included gender, level of education, experience, position, participation in GBs, and number of GBs accomplished. Table 8 depicts the demographic characteristics of respondents.

**Table 8:** Demographic characteristics of respondents.

Category	Classification	Frequency	Percentage
Gender	Male	81	65.85
	Female	42	34.15
Level of education	Diploma	5	4.07
	Bachelor	74	60.16
	Master	41	33.33
	PhD	3	2.44
Experience	1–5 years	32	26.02
	11–20 years	29	23.58
	5–10 years	59	47.97
	Less than 1 year	3	2.44
Position	Quantity surveyors	4	3.25
	Architects	4	3.25
	Clients	4	3.25
	Site engineers	50	40.65
	Technicians	29	23.58
	Facility manager	4	3.25
	Consultant	4	3.25
	Site managers	4	3.25
	Environmental experts	4	3.25
	Contractor representatives	16	13.01
GB participation	Almost always	1	0.81
	Never	105	85.37
	Often	5	4.07
	Seldom	7	5.69
	Sometimes	5	4.07

### 4.3. Awareness Level of I4.0 Technologies in GBs

The first objective of the study was to assess the awareness level regarding the I4.0 technologies on GBs; the first part of awareness was categorized as assessing the overall familiarity with terminologies related to I4.0, GBs and availability of I4.0, then the study assessed the awareness of I4.0 technologies, and finally, the awareness of which I4.0 technologies can be applied in GB was assessed.

#### 4.3.1. Familiarity With Terminologies

Respondents were asked to rate the questions, asking if they were familiar with I4.0, the study assessed whether they were aware of the available I4.0 technologies, and finally, respondents were asked if they were familiar with GB. The awareness level was assessed based on the model used by Eski et al. [58] to assess students′ awareness of winter sports on a points Likert scale. The Eski et al.′s [58] scale used considered the mean ratings for the questions assessing awareness and categorized them into five awareness ranges, as shown in Figure 2.

The findings on the awareness regarding I4.0 terminologies awareness level revealed that IG3 “Are you familiar with the term GBs” was the terminology which respondents were highly aware of, whereas for IG1 “Are you familiar with the term I4.0” and IG2 “Are you familiar with the available I4.0 technologies” had a medium level of awareness among the respondents as shown in Table 9. As it was for individual items, the overall awareness level was also found to be medium, with a mean score of 3.15. Also, the awareness level was analyzed by determining the RII. The awareness item with a high RII score was IG3 (0.710), whereas the lowest was IG2 (0.568), as shown in Table 9. The findings indicate that both mean scores and RII scores implied the same results.

**Table 9:** Descriptive statistics on the awareness level of I4.0 terminologies.

Awareness items	Mean	Std. deviation	RII	Level of awareness
IG1	3.06	1.237	0.613	Medium level of awareness
IG2	2.84	1.098	0.568	Medium level of awareness
IG3	3.55	1.121	0.710	High level of awareness

#### 4.3.2. Awareness Level of I4.0 Technologies in GBs

The next step in the study was to determine how much respondents knew about I4.0 technologies. The results of the awareness assessment showed that, among all I4.0 technologies, AI was the most well‐understood, with a mean rating of 4.68, which is considered complete awareness on Eski et al.′s [58] awareness scale. On the other hand, BIM also fell under the complete awareness level, with a score of 4.42. With mean ratings of 4.06 and 3.97, respectively, cybersecurity and the IoT were the two technologies with high awareness. The other I4.0 technologies had a medium level of awareness, with AR having the lowest mean rating of 2.68, as summarized in Table 10. The overall awareness level for all I4.0 technologies was found to be 3.31, which indicates a medium level of awareness.

**Table 10:** Awareness of I4.0 technologies in GB.

Code	I4.0 technologies	Mean	Std. deviation	RII	Awareness level
AT1	AI	4.68	0.541	0.935	Complete level
AT2	CPSs	3.06	1.181	0.613	Medium level
AT3	BDAs	2.71	1.071	0.542	Medium level
AT4	Autonomous robotics	3.03	1.251	0.606	Medium level
AT5	Cyber security	4.06	0.629	0.813	High level
AT6	BIM	4.42	0.502	0.884	Complete level
AT7	IoT	3.97	0.983	0.794	High level
AT8	CC	3.19	1.108	0.639	Medium level
AT9	Additive manufacturing (3D printing)	3.29	1.071	0.658	Medium level
AT10	AR	2.68	1.077	0.535	Medium level
AT11	Cyber manufacturing	2.71	0.973	0.542	Medium level
AT12	VR	3.16	1.157	0.632	Medium level
AT13	BT	2.74	1.094	0.548	Medium level
AT14	Radio frequency identification (RFID)	2.71	1.189	0.542	Medium level

#### 4.3.3. Awareness of Principles of GBs

The awareness level of the five principles of GBs was assessed, and the result showed that respondents were completely aware of two principles (energy efficiency and waste reduction) with a mean rating of 4.48 and 4.32, respectively. In contrast, high‐level awareness was observed for indoor environmental quality with a mean rating of 3.87, water conservation with a mean rating of 3.84, and material selection with a mean rating of 3.81, as seen in Table 11. The overall mean score for the principles of GBs was 4.06, indicating a high level of awareness.

**Table 11:** Awareness level on principles of GBs.

Code	Principle	Mean	Std. deviation	RII	Awareness level
PGB1	Energy efficiency	4.48	0.677	0.897	Complete level
PGB2	Water conservation	3.84	0.898	0.768	High level
PGB3	Material selection	3.81	1.078	0.761	High level
PGB4	Indoor environmental quality	3.87	0.957	0.774	High level
PGB5	Waste reduction	4.32	0.748	0.865	Complete level

#### 4.3.4. I4.0 Technologies Application in GBs

After asking respondents which I4.0 technologies they considered useful in Tanzania′s GBs, the responses were descriptively analyzed using the mean scores and RII to determine which technologies were more crucial to use in GBs than others. The findings indicated that the most significant technology that might be used in GB was BIM, which had a high mean rating (4.58) and RII (0.916). BDAs ($\bar{\text{X}}$ = 4.45, RII = 0.890), additive manufacturing ($\bar{\text{X}}$ = 4.26, RII = 0.852), AI ($\bar{\text{X}}$ = 4.03, RII = 0.806), and CC ($\bar{\text{X}}$ = 4.03, RII = 0.806) were the other four highly ranked technologies that made up the top five. Cyber manufacturing had the lowest rating ($\bar{\text{X}}$ = 2.81, RII = 0.561). Table 12 shows the results. The overall awareness of I4.0 technologies was found to be high, with a mean score of 3.53.

**Table 12:** I4.0 technologies for GB.

Code	I4.0 technologies	Mean	Std. deviation	RII
IT1	AI	4.03	0.983	0.806
IT2	CPSs	2.84	0.969	0.568
IT3	BDAs	4.45	0.925	0.890
IT4	Autonomous robotics	3.10	1.248	0.619
IT5	Cyber security	3.39	1.174	0.677
IT6	BIM	4.58	0.620	0.916
IT7	IoT	3.77	0.845	0.755
IT8	CC	4.03	1.224	0.806
IT9	Additive manufacturing (3D printing)	4.26	1.154	0.852
IT10	AR	2.87	1.024	0.574
IT11	Cyber manufacturing	2.81	1.014	0.561
IT12	VR	3.13	1.147	0.626
IT13	BT	2.87	1.118	0.574
IT14	RFID	3.26	1.154	0.652

### 4.4. GB′s Environmental Performance

**Figure 3:**
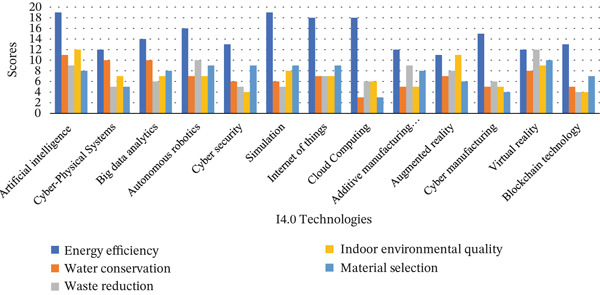
Applicability of I4.0 technologies in GB principles.

The study assessed the usefulness of I4.0 technologies in GB principles; respondents were asked to indicate which technologies were useful in each GB principle. The analysis results revealed that energy efficiency was the GB principle in which all I4.0 technologies were highly employed, with at least 38.7% of respondents mentioning energy efficiency for all I4.0 technologies. Figure 3 depicts the applicability of I4.0 technologies in GB principles.

The study collected data from the four GB projects. The results for the actual measured outcomes (energy efficiency, water conservation, material selection, indoor environmental quality, and waste reduction) are shown in Table 13. The study found that the I4.0 technologies are projected to enhance energy efficiency ( 15%–25%), water conservation (≥ 20%), improve indoor environmental quality, improve material selection, and lead to waste reduction (Table 14).

**Table 13:** The results before the green certification versus the projected or measured green outcomes.

Principle	Before green certification				Projects or measured green outcomes			
	GBP1	GBP2	GBP3	GBP4	GBP1	GBP2	GBP3	GBP4
Energy efficiency	Standard grid electricity uses with conventional HVAC/lighting	Conventional building materials	Typical Tanzanian housing	Conventional interior lighting and HVAC	≈10% lower electricity use annually due to energy‐efficient lighting and MSS controlling solar heat gain and cooling loads	Projected 20% reduction in energy use	Significant energy reduction through passive design (orientation, natural ventilation, daylighting)	Projected ≥ 15%–20% reduction via LED lighting, sensors, efficient HVAC for interior spaces
Water conservation	Standard fixtures with efficiency measures	Conventional fixtures	Conventional plumbing	Standard fixtures	Projected reduction via low‐flow fixtures and efficient plumbing design	Projected 20% reduction in water usage	Projected 20%–30% reduction in water usage	Projected ≥20% water savings through efficient fixtures and monitoring
Material selection	Conventional materials with no sustainability criteria	Standard materials	Standard bricks or concrete	Standard interior materials	Use of selected low‐impact and eligible materials	Use sustainable building materials with lower embodied impacts	Use compressed stabilized earth blocks (CSEBs), bamboo and other recycled materials	Low‐emitting materials and recycled content are used in finished products and furnishings
Indoor environmental quality	Standard ventilation and daylighting	Standard indoor conditions	Standard housing conditions	Standard indoor conditions	Improved IEQ due to enhanced ventilation strategies, daylight access and reduced glare	Expected improvement through design criteria (ventilation, materials with low VOC)	Improved IEQ due to enhanced ventilation strategies, daylight access, and reduced glare	Improved air quality, ventilation, and lighting comfort consistent with IEQ credits
Waste reduction	Site and construction waste are unmanaged	No formal waste strategy	Conventional building waste	Conventional interior construction waste	Waste management plan required to be at least 50% construction waste diverted from landfill (projected)	Waste management during construction and operations improved; projected moderate waste diversion	On‐site prefabrication and local sourcing of some materials, including soil materials	Construction waste management plan implemented; the project can typically manage to achieve ≥ 50% diversion (projected)

Abbreviation: GBP, green building project.

**Table 14:** Summary comparison table of the green building principles.

Green project	Energy efficiency	Water conservation	Material selection	Indoor environmental quality	Waste reduction
GBP1	≈10% (measured)	≥ 15%–25% (projected)	Improved specifications	Improved	≥ 50% diversion (projected)
GBP2	≥ 20% (projected)	≥ 20% (projected)	Better materials	Better	Moderate (projected)
GBP3	High passive	≥20%–30% (projected)	Low embodied energy	Strong natural IEQ	Zero‐waste construction
GBP4	≥15%–25% (projected)	≥ 20% (projected)	Low‐emitting materials	Enhanced IEQ	≥ 50% diversion (projected)

### 4.5. Challenges of GB Implementation

The study assessed challenges that impose difficulty in implementing GBs in Tanzania; a descriptive analysis of the challenges was performed, followed by the normalization of the mean technique used by Mukhametzyanov [61]. Normalization of means was used to determine which challenges were critical in the hindrance of GB implementation in Tanzania. The criticality is obtained through normalization of the mean score by the formula (mean‐minimum mean/maximum mean‐minimum mean); the normalization values greater than 0.5 for any item, such an item is considered critical [61]. As shown in Table 15, only four challenges were considered as not critical; “Lack of Standardization and Certification” with 0.176 normalization value, “Culture and Aesthetics” with a 0.000 normalization value, “Client demand” with 0.135 and “Regulation and Policy” with normalization value of 0.297. All other challenges were considered critical, whereas “Allocated funds to support” was the most critical challenge, with a normalization value of 1.000.

**Table 15:** Criticality of challenges for implementing GB.

Challenges	Mean	Std. deviation	Normalization	Criticality
High initial cost	4.06	1.031	0.689	Critical
Awareness and education	4.16	0.86	0.824	
Infrastructure	4.00	0.931	0.608	
Allocated funds to support	4.29	0.973	1.000	
Lack of knowledge and skills	4.16	0.898	0.824	
Availability of materials and technologies	3.94	0.892	0.527	
Lack of standardization and certification	3.68	1.077	0.176	Not critical
Culture and aesthetics	3.55	1.121	0.000	
Client demand	3.65	0.877	0.135	
Regulation and policy	3.77	1.146	0.297	

### 4.6. Factors for Applying I4.0 Technologies in GBs

This section analyzed responses on the factors that influence the applicability of I4.0 technologies in GBs. These factors were categorized into seven variables, each with factors within it: technology, top management commitment, mission/vision, employee skills, infrastructure, financial arrangement, and environmental factors. The first part of the analysis on factors included EFA to align factors with their respective variables, then the influence of factors for applying I4.0 technologies in GBs was assessed through regression analysis.

#### 4.6.1. EFA of Factors

Components were extracted using EFA in SPSS 21.0. The EFA was performed using factor loadings more than 0.5 for each factor on its variables, and components were recovered using eigenvalues greater than one [59]. Compared with the seven variables included in the literature study, the factors′ analysis yielded six components with eigenvalues greater than one. The EFA results show that components under top management commitment and mission or vision were consolidated into a single variable, indicating that they were all measuring the same construct; hence, the mission or vision variable was discarded. Three factors were eliminated: “TC4—autonomous robotics,” “TC11—cyber manufacturing,” and “TC13—blockchain technology” (Table 16). These factors were removed based on the results of the factor analysis, where they exhibited factor loadings below the acceptable threshold (0.50) and did not load significantly onto any of the extracted components. In addition, these items showed weak communalities, indicating that they did not share sufficient variance with other variables in the model. As such, their inclusion would have reduced the clarity and interpretability of the factor structure. In fact, the decision to eliminate these items is consistent with established practices in EFA, where variables that fail to meet minimum loading criteria or do not align with any factor are removed to improve model robustness. Furthermore, no substantial cross‐loadings were observed for these variables, reinforcing their lack of meaningful contribution to the extracted components. Likewise, regarding construct validity, the removal of these items does not weaken the measurement model; rather, it enhances it by ensuring that only variables with strong and meaningful associations are retained. The refined factor structure demonstrates improved convergent validity, as retained items load significantly on their respective components, and better internal consistency across constructs. This refinement results in a more reliable and theoretically coherent representation of I4.0 adoption factors in the context of GBs.

**Table 16:** Factors influencing applicability of I4.0 in GBs.

Component	Items	Component					
		1	2	3	4	5	6
Technology	TC1	0.806					
	TC2	0.852					
	TC3	0.782					
	TC5	0.731					
	TC6	0.839					
	TC7	0.815					
	TC8	0.686					
	TC9	0.931					
	TC10	0.789					
	TC12	0.896					
Top management commitment	TMC1		0.957				
	TMC2		0.804				
	TMC3		0.911				
	MV1		0.852				
	MV2		0.882				
	MV3		0.723				
Employee skills	ES1			0.655			
	ES2			0.866			
	ES3			0.805			
	ES4			0.794			
Infrastructure	IF1				0.874		
	IF2				0.781		
	IF3				0.782		
Financial arrangement	FA1					0.873	
	FA2					0.789	
	FA3					0.798	
	FA4					0.7366	
Environmental factors	EF1						0.863
	EF2						0.881
	EF3						0.681

*Note:* Extraction method: principal component analysis; rotation method: Varimax with Kaiser normalization. Rotation converged in 7 iterations.

**Figure 4:**
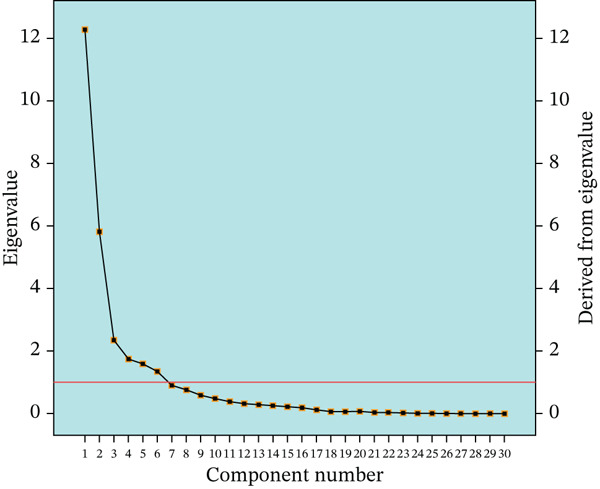
Eigenvalues for component extraction.

Figure 4 shows the eigenvalues for the components extracted from the factor for applying I4.0 in GBs in Tanzania. Only six components had eigenvalues greater than one; hence, they were extracted. The six extracted components had a cumulative percentage of variance explanation of 83.709, as shown in Table 17.

**Table 17:** Percentage variance of explanation of extracted components.

Component	Initial eigenvalues			Extraction sums of squared loadings			Rotation sums of squared loadings		
	Total	% of variance	Cumulative %	Total	% of variance	Cumulative %	Total	% of variance	Cumulative %
1	12.272	40.908	40.908	12.272	40.908	40.908	7.236	24.120	24.120
2	5.827	19.424	60.332	5.827	19.424	60.332	5.663	18.876	42.997
3	2.341	7.805	68.137	2.341	7.805	68.137	3.431	11.437	54.434
4	1.744	5.813	73.949	1.744	5.813	73.949	3.077	10.256	64.690
5	1.580	5.267	79.217	1.580	5.267	79.217	2.945	9.816	74.507
6	1.348	4.493	83.709	1.348	4.493	83.709	2.761	9.203	83.709
7	0.910	3.032	86.741						
8	0.769	2.565	89.306						
9	0.594	1.980	91.286						
10	0.474	1.581	92.867						
11	0.380	1.268	94.136						
12	0.329	1.096	95.232						
13	0.293	0.978	96.209						
14	0.258	0.860	97.069						
15	0.217	0.722	97.791						
16	0.193	0.642	98.433						
17	0.121	0.404	98.837						
18	0.081	0.269	99.106						
19	0.069	0.230	99.337						
20	0.066	0.218	99.555						
21	0.047	0.157	99.712						
22	0.030	0.100	99.812						
23	0.023	0.075	99.887						
24	0.015	0.049	99.936						
25	0.012	0.041	99.978						
26	0.005	0.018	99.995						
27	0.001	0.003	99.999						
28	0.000	0.001	100.000						
29	1.002E‐013	1.008E‐013	100.000						
30	1.001E‐013	1.004E‐013	100.000						

*Note:* Extraction method: principal component analysis.

### 4.7. Assessing the Influence of Factors for Applying I4.0 to GBs in Environmental Performance

This section assessed how factors for applying I4.0 technologies in GBs influence environmental performance. This was done by conducting linear regression analysis in SPSS 21.0. A linearity test was conducted, followed by a regression analysis.

#### 4.7.1. Linearity Test

In order to carry out linear regression analysis, it is necessary to operate under the assumption that the dependent variables and the independent variables are linearly related [60]. It was necessary to carry out the linearity test in order to guarantee that the variables had a linear connection with one another. The significant values of the departure from linearity in Table 18 demonstrated that the linearity test was successful in establishing a linear connection between all of the independent factors and the dependent variable. The independent variable is considered linearly related to a dependent variable if it has significant values for the deviation from the mean greater than 0.05 [60].

**Table 18:** Linearity test results between dependent and independent variables.

Variables		Sum of squares	df	Mean square	F	Sig.
Environmental performance ∗ technology	(Combined)	18.141	13	1.395	2.514	0.038
	Linearity	11.173	1	11.173	20.13	0.001
	Deviation from linearity	6.967	12	0.581	1.046	0.455
Environmental performance ∗ top management commitment	(Combined)	17.642	16	1.103	1.554	0.207
	Linearity	2.707	1	2.707	3.815	0.041
	Deviation from linearity	14.934	15	0.996	1.403	0.266
Environmental performance ∗ employee skills	(Combined)	9.815	10	0.982	1.105	0.404
	Linearity	3.281	1	3.281	3.694	0.039
	Deviation from linearity	6.535	9	0.726	0.818	0.607
Environmental performance ∗ Infrastructure	(Combined)	10.414	9	1.157	1.416	0.244
	Linearity	6.76	1	6.76	8.271	0.009
	Deviation from linearity	3.654	8	0.457	0.559	0.799
Environmental performance ∗ Financial	(Combined)	8.515	11	0.774	0.772	0.043
	Linearity	0.226	1	0.226	0.225	0.041
	Deviation from linearity	8.289	10	0.829	0.826	0.609
Environmental performance ∗ Environmental factors	(Combined)	16.538	8	2.067	4.12	0.004
	Linearity	12.082	1	12.082	24.078	0.001
	Deviation from linearity	4.456	7	0.637	1.269	0.310

#### 4.7.2. Regression Analysis on Factors

Six independent variables (technology, top management commitment, staff skills, infrastructure, financial arrangement, and environmental elements) and one dependent variable (environmental performance) were subjected to multiple regression analysis using SPSS 21.0. The relationship between a single dependent variable and predictor variables is examined using regression analysis [65]. The ability of the regression line to explain all of the variations in the dependent variable is shown in the model summary table. Table 19 provided *R* and *R* ^2^ values. *R*‐value (0.723) represents a simple correlation, indicating a high degree of correlation *R* ^2^ shows the extent to which the predictor variables technology, top management commitment, staff skills, infrastructure, financial arrangement, and operational control factors can account for the whole variation in the dependent variable (environmental factors). In fact, the regression shows associations, not proof that I4.0 causes improved environmental performance. It is suggested to perform longitudinal studies to track actual I4.0 adoption over time. In this instance, 52.3% can be explained. Table 20 provides an overview of the findings of the ANOVA test, which was used to determine the model′s significance. The mean square value was obtained by dividing the sum of squares by the degree of freedom.

**Table 19:** Model Summary.

Model	*R*	*R*square	Adjusted *R* squar	Std. error of the estimate
1	0.723^a^	0.523	0.499	0.67199

^a^Predictors: (constant), operational control factors, financial arrangement, infrastructure, employee skills, top management commitment, and technology.

**Table 20:** Analysis of variance.

Model		Sum of squares	df	Mean square	*F*	Sig.
1	Regression	57.505	6	9.584	21.224	0.004
	Residual	52.382	116	0.452		
	Total	109.887	122			

*Note:* Dependent variable: Environmental performance. Predictors: (constant), operational control factors, financial arrangement, infrastructure, employee skills, top management commitment, technology.

Based on the results in Table 20, the values when computing mean square (model/between groups) were 9.584. This implies that the mean square values are good because the independent variables (predictors) have a strong effect, or that the groups differ significantly from each other. Likewise, the value when computing the mean square (error/residual/within), its score was 0.452, indicating that the score was excellent because when its value is closer to 0, that is a perfect score. In fact, a lower value indicates that the model′s predictions are close to the actual values and that the data regarding the application of I4.0 technologies for GB projects are not widely spread out.

The findings in Table 20, presented statistically, indicate that the regression model generates a significant number of precise predictions for the dependent variable. Table 20 demonstrates the current statistical significance of the model. The regression model has statistically significant predictive capability for the outcome variable, shown by a *p* value of 0.004, which is below the 0.05 threshold. This indicates that the model aligns well with the data. The F critical value at the 5% significance level, as per the standard *F*‐tables, is 2.55. The estimated *F* value (21.224) exceeds the crucial *F*‐value, indicating that the overall model is significant [66].

In this study, the regression analysis tested six hypotheses for each independent variable′s significant impact on the environmental performance of GBs. The coefficients that are not standardized illustrate the degree to which the dependent variable varies in response to an independent variable, supposing that all of the other independent variables stay unchanged. Table 21 shows the coefficients of regression.

**Table 21:** Regression coefficient.

Model		Unstandardized coefficients		Standardized coefficients	*t* *-*value	*p*	Collinearity statistics	
		B	Std. error	Beta			Tolerance	VIF
1	(Constant)	0.450	0.067		6.722	< 0.0001		
	Technology	0.328	0.117	0.277	2.801	0.0059	0.421	2.377
	Top management	0.200	0.101	0.118	1.976	0.0504	0.469	2.131
	Employee skills	0.422	0.114	0.059	3.699	0.0003	0.580	1.723
	Infrastructure	0.159	0.023	0.161	6.916	0.0001	0.581	1.721
	Financial	0.545	0.113	0.074	4.819	0.0001	0.506	1.976
	Operational control	0.481	0.119	0.387	4.041	0.0001	0.448	2.232

*Note:* Dependent variable: environmental performance of GBs.

The factors for applying I4.0 to GBs for enhancing environmental performance are as follows. The technology involved ranking subtechnologies such as AI, CPSs, BDAs, autonomous robotics, cybersecurity, simulation, the industrial IoT, CC, additive manufacturing (3D printing), AR, cyber manufacturing, VR, and BT. Top management commitment involved subfactors in encouraging the use of I4.0, assessing knowledge on the use of I4.0 technologies, and ranking the support and engagement in utilizing I4.0 technologies.

Mission or vision and strategy involved assessing the presence of projects′ vision and strategy aligned with the I4.0 concept and the plans or processes for implementing I4.0 in enhancing environmental performance. Employee skills and adaptability factors were checked based on knowledge of I4.0 technologies, skills in applying I4.0 technologies, competence to use I4.0 technologies, and whether the GB project recognized the benefits of implementing I4.0 technologies. The infrastructure factor looked at available ICT systems and the reliable internet to support I4.0. Financial arrangements involved the availability of financial resources, budget allocation, financial software to manage GBs, and an I4.0‐specific budget. Operational control factors involved looking at the applicability of I4.0 for enhancing environmental sustainability, I4.0 technologies deployment and integrating I4.0 technologies to enhance environmental sustainability in the GB.

In view of the results in Table 21 generated from SPSS output, the regression equation extracted was as presented in Equation (2).

(2) Y=0.4500.32810.20020.42230.15940.54550.4816+X+X+X+X+X+X+Ɛ.

As demonstrated by *β* = 0.328, *p* ≤ 0.0059, technology is found to be statistically significant in explaining the successful effect on environmental performance of GBs. Given that the *p* value (0.0059) is smaller than the chosen level of significance (0.05), the influence is significant. This suggests that technology has a major and positive impact on environmental performance. As a result, advancements in technology will result in a 0.328‐unit improvement in environmental performance.

Additionally, as indicated by *β* = 0.200, *p* = 0.0504, the results demonstrated that top management commitment is thought to be statistically significant in explaining the environmental performance of GBs. Given that the *p* value (0.0504) is below the chosen level of significance (0.05), the influence is significant. This suggests that the environmental performance of GBs is positively and significantly influenced by top management commitment. Consequently, an increase in top management commitment will result in a 0.200‐unit improvement in the environmental performance of GBs.

It has been observed that the abilities of employees have a statistically significant impact on the effective influence of GBs on environmental performance, as shown by the coefficient (*β*) of 0.422 and the *p* value of 0.0003. Due to the fact that the *p* value (0.0003) is lower than the threshold of significance that was chosen (0.05), the impact is considered to be significant. It may be deduced from this that environmental performance is greatly and favorably influenced by the abilities of the employees. Therefore, increasing the abilities of employees will result in a 0.422‐unit improvement in the performance of the environment.

According to *β* = 0.159, *p* = 0.0001, infrastructure is found to be statistically significant in describing how well GBs influence their environmental performance. Given that the *p* value (0.0001) is below the chosen level of significance (0.05), the influence is significant. This suggests that environmental performance is positively and significantly influenced by infrastructure. Thus, there will be a 0.159‐unit increase in environmental performance as a result of improved infrastructure.

As demonstrated by *β* = 0.545, *p* = 0.0001, financial arrangements are seen to be statistically significant in explaining the successful environmental performance of GBs. Given that the *p* value (0.0001) is smaller than the chosen level of significance (0.05), the influence is significant. This suggests that financial arrangements have a positive and significant impact on the environmental performance of GBs. Consequently, a 0.545‐unit improvement in environmental performance will result from increased financing.

As shown by the statistically significant coefficient (*β* = 0.481, *p* = 0.0001), operational control components play a significant role in revealing the powerful impact that GBs have on the environmental performance of the structures. Given that the *p* value (0.0001) is lower than the predetermined significance criterion (0.05), the impact seems to be rather significant. According to this, the characteristics of operational control have a positive and significant influence on the performance of the environment. Furthermore, technological developments will lead to an improvement in environmental performance that is equal to 0.481 units.

### 4.8. Strategies for Implementation of I4.0 Technologies in GBs

The strategies for implementing I4.0 in GBs were assessed in this section, and the responses were subjected to descriptive analysis in SPSS 21.0 (Table 22). The mean rating (mean = 4.48) for the strategies revealed that ST4 “Emphasizing automation, IT infrastructures and technologies within the companies” was the highest rated strategy for implementing I4.0 in GBs, ST13 “The government set up policies and plans for I4.0 technologies” was the second highest rated strategy with a mean score of 4.38. The other three factors for the top five highly rated strategies were ST5 “Investment in new technologies” (mean = 4.35), ST9 “Knowledge of the needed hardware” (mean = 4.35), and ST3 “Collaboration and partnership on digital technologies” (mean = 4.32).

**Table 22:** Action strategies for implementing I4.0 Technologies in GBs.

Code	Strategy category	Action strategy	Operationalization	Mean	Std. deviation	Communalities
ST1	A clear vision of digital strategy;	Develop a national and organizational digital transformation roadmap aligned with green building standards.	• Establish a digital transformation unit within construction firms.	4.29	0.661	0.679
			• Conduct baseline digital maturity assessments.			
			• Align strategy with Tanzania′s smart city initiatives.			
			• Integrate I4.0 into existing construction and sustainability policies.			
			• Set measurable KPIs (e.g., energy efficiency %, carbon reduction, smart system adoption rate).			
ST2	Education and training;	Build capacity in I4.0 technologies such as IoT, BIM, AI, and automation.	• Partner with universities and technical colleges to create certified programs in smart construction.	4.22	0.633	0.878
			• Provide on‐site training and workshops for engineers and technicians.			
			• Introduce continuous professional development (CPD) requirements.			
ST3	Collaboration and partnership on digital technologies;	Foster multistakeholder collaboration between government, private sector, and academia.	• Create innovation hubs or clusters focused on smart and green construction.	4.32	0.622	0.883
			• Establish partnerships with international tech firms for knowledge transfer.			
			• Encourage public–private partnerships (PPPs) for pilot smart building projects.			
ST4	Emphasizing automation, IT infrastructures and technologies within the companies;	Integrate automation systems and digital tools into building lifecycle processes.	• Deploy building information modeling (BIM) across projects.	4.48	0.547	0.66
			• Install IoT‐based energy management systems in buildings.			
			• Digitize construction workflows (e.g., cloud‐based project management tools).			
ST5	Investment in new technologies;	Promote targeted investments in smart and sustainable technologies.	• Offer tax incentives or subsidies for adopting green digital technologies.	4.35	0.632	0.784
			• Encourage firms to allocate R&D budgets for innovation.			
			• Pilot projects showcasing smart green buildings in major cities.			
ST6	Costs and issues associated with the implementation of I4.0 solutions;	Address financial and technical barriers to I4.0 adoption.	• Develop cost‐sharing models (e.g., leasing smart equipment).	4.32	0.567	0.807
			• Provide low‐interest loans or grants for SMEs.			
			• Conduct cost‐benefit analyses before implementation.			
ST7	Clear benefits from digital investments;	Demonstrate measurable value from I4.0 adoption.	• Document case studies showing energy savings and ROI.	4.26	0.596	0.722
			• Use dashboards to track performance metrics (energy use, maintenance costs).			
			• Share success stories across the industry.			
ST8	Advantages related to data security, privacy, and responsibility;	Ensure secure and ethical use of digital systems.	• Develop and enforce data governance policies.	4.19	0.617	0.864
			• Implement cybersecurity frameworks for smart buildings.			
			• Train staff on data protection and compliance standards.			
ST9	Knowledge of the needed hardware;	Improve awareness of required digital infrastructure.	• Create guidelines/standards for hardware selection (sensors, smart meters, and servers).	4.35	0.578	0.689
			• Engage suppliers to provide technical demonstrations.			
			• Develop procurement frameworks for quality assurance.			
ST10	Sufficient funds to invest in digitalized equipment;	Ensure financial readiness for digital transformation.	• Establish dedicated digital transformation budgets.	4.32	0.672	0.641
			• Leverage international funding (e.g., climate finance, development banks).			
			• Promote green bonds and investment schemes.			
ST11	Reliable internet and electricity;	Strengthen enabling infrastructure.	• Collaborate with telecom providers to ensure high‐speed connectivity on construction sites.	4.22	0.58	0.805
			• Integrate renewable energy solutions (e.g., solar backup systems).			
			• Advocate for infrastructure upgrades in urban development plans.			
ST12	Skilled workforce;	Build a digitally competent construction workforce.	• Develop skill certification frameworks for I4.0 competencies.	4.22	0.58	0.893
			• Encourage internships and apprenticeships in smart construction projects.			
			• Attract diaspora and international experts for knowledge transfer.			
ST13	The government set up policies and plans for I4.0 technologies;	Develop integrated policies and regulatory frameworks that explicitly support the adoption of I4.0 technologies in the construction and GBs	• Tax incentives for smart and GBs technologies.	4.38	0.53	0.925
			• Import duty reductions on digital construction equipment.			
			• Grant grants for pilot smart building projects.			
			• Track policy effectiveness and adjust dynamically.			

The analysis for the proposed strategies involved the computation of mean, standard deviation and the PCA in SPSS 21.0. The PCA was performed by testing the communalities (*h* ^2^), whereby such communalities (*h* ^2^) signify the proportion of each observed variable′s total variance that is explained by the retained underlying factors. When there are higher values, this indicates that the factor model well‐represents the variable, whereas low values suggest the variable is mostly unique or error variance. Based on the results in Table 20, the lowest value was for ST10 (0.641), followed by ST4 (0.660). Such results imply that the proposed strategies are acceptable because they all achieved the communalities of at least 0.50 [67]. The computation of the communalities involves the consideration of the sum of the squared factor loadings for a variable across all extracted factors. So, for ST13, this means there is a communality of 0.925, which means 92.50% of that strategy′s variance is explained by the common factors. If the value were to be less than 0.50, such a strategy was not fitting the selection, thus it could have been removed due to its low communalities. Thus, as a result of both descriptive analyses with the mean rating between 4.19 and 4.48 and the communality values between 0.591 and 0.875, all strategies were found to be reliable for implementing I4.0 in GBs.

### 4.9. Linkage Between I4.0 and GBs

The results have demonstrated the positive linkage between I4.0 and GBs, mainly on how I4.0 affects the environmental performance of GBs. For example, from Table 20, it is observed that *β* = 0.328, *p* = 0.0059. This means that technology is found to be statistically significant in explaining the successful effects of GBs on environmental performance. Given that the *p* value (0.0059) is smaller than the chosen level of significance (0.05), the influence is significant. This suggests that technology has a major and positive impact on environmental performance. As a result, advancements in technology will result in a 0.328‐unit improvement in environmental performance [68]. Similar findings were observed in other studies. For example, Siekmann et al. [69] concluded that there is a positive linkage between sustainability, mainly for GBs, and I4.0. Likewise, Hübner et al. [70] found a positive linkage when their study identified different types of implemented I4.0 technologies in three green‐rated star buildings and compared the level of energy consumption.

I4.0 strategy positively impacts environmental and economic sustainability [25]. The strategy can be applied to another context. Inform policy development for green construction. The strategy focuses on the construction phase. The implementation of the strategy is hindered by technology adoption due to cultural and personnel skills [25].

Moreover, Chen et al. [19] argued that as it encompasses the idea of sustainable development, the smart city now provides the ideal condition for urban growth. BIM is becoming a vital tool for the construction industry, and C4.0, which has its roots in I4.0, is just two of the cutting‐edge ideas and technologies being integrated into the construction industry to create a smart city. Thus, in the smart city context, this article seeks to examine the state of the art and future directions of the multidisciplinary integration of C4.0, I4.0, BIM, and I4.0 technologies, which have the potential to transform the construction industry, and GBs are part of it [32]. A CPS strategy is proposed to integrate I4.0 technologies and improve construction capabilities. The strategy leverages IoT to connect the physical construction site with the cloud‐based cyber part, enabling real‐time data exchange and feedback loops for improved monitoring, decision‐making, and collaboration. The strategy enables real‐time construction site monitoring, allowing for proactive decision‐making and adjustments based on current data [19]. Implementing this strategy requires significant upfront investment in technology infrastructure, software, and training, which might not be feasible for all construction organizations.

It can finally be observed that I4.0 is important in influencing environmental performance for GBs. This is because the *R* and *R* ^2^ values of the regression model suggest so. For example, *R*‐value (0.723) represents a simple correlation, indicating a high degree of correlation *R* ^2^ shows how much the predictor variables technology, top management commitment, staff skills, infrastructure, financial arrangement, and operational control factor can account for the variation in the dependent variable (environmental performance of GBs). *R* ^2^ value was found to be 0.523, thus indicating that the independent variables can explain 52.3% of the variance in the dependent variable. A model with an *R*‐squared above 0.50 is good, provided that some or most of the explanatory variables are statistically significant [71].

### 4.10. Significance of the Study

GBs are essential because they can minimize the negative effects on the environment. Integrating green features into the design and construction of the building increases the efficient use of water, energy, and materials. The application of I4.0 in GBs is still low, but it has the potential to change the environment. Many processes involved in the construction of GBs through I4.0 tend to lower costs while increasing productivity. Collecting and analyzing GBs data is relevant to improving the buildings′ design features and increasing their performance. This study also contributes to the effective performance of GBs in Tanzania, particularly in Dar es Salaam. I4.0 serves as the foundation for transforming how building projects operate and introducing real digital approaches in building construction.

## 5. Conclusion and Recommendations

### 5.1. Conclusion

The construction industry is increasingly drawing attention in many parts of the world to apply I4.0‐related technologies. The installation of I4.0‐related technologies captures the attention of construction activity as robotics and machines can operate in the construction sector. The construction industry involves the presence of GBs. GBs tend to expand the classical design to improve the economy, utility, and create comfort. It refers to saving resources for maximum energy, land, water, and material savings. Therefore, this research focused on assessing the applicability of I4.0 technologies on GBs for enhancing environmental performance in Dar es Salaam, as one of the major cities with four GB projects. Chiefly, the research considered three specific objectives. First, to determine the awareness level of I4.0 technologies on GBs; second, to determine factors for applying I4.0 technologies to GBs; and lastly, to propose I4.0 strategies for GBs for enhancing environmental performance. The research focused on four completed GB projects in Tanzania that are LEED‐certified by USGBC.

The next step in the study was to determine how much respondents knew about I4.0 technologies. The results of the awareness assessment showed that, among all I4.0 technologies, AI was the most well‐understood, with a mean rating of 4.68, which is considered complete awareness on the awareness scale (1 to 5). BIM also fell under the complete awareness level, with a score of 4.42. With mean ratings of 4.06 and 3.97, respectively, cybersecurity and the IoT were the two technologies with high awareness. The other I4.0 technologies had a medium level of awareness, with AR having the lowest mean rating of 2.68. The overall awareness level for all I4.0 technologies was found to be 3.31, which indicates a medium level of awareness. In general, GBs in Tanzania were found to be applying some I4.0 technologies, and the awareness level for the majority of I4.0‐related technologies was found to be high, ranging from 2.68 to 4.68 (on a scale of 1–5).

The assessment of awareness levels regarding the five principles of GBs revealed that respondents were fully aware of energy efficiency (mean rating of 4.48) and waste reduction (mean rating of 4.32). Additionally, high awareness was noted for indoor environmental quality (3.87), water conservation (3.84), and material selection (3.81). The overall mean score for GB principles was 4.06, indicating a strong awareness level.

The study also assessed challenges that pose difficulties in implementing GBs in Tanzania. The challenges were analyzed through the descriptive analysis, followed by the normalization of the mean technique. Normalization of means was used to determine which challenges were critical in the hindrance of GB implementation in Tanzania. The criticality is obtained through normalization of the mean score by the formula (mean score minus minimum mean/maximum mean minus minimum mean), and whenever the normalization values are greater than 0.5 for any item, such an item is considered critical. So, only four challenges were considered as not critical: “lack of standardization and certification” with a 0.176 normalization value, “culture and aesthetics” with a 0.000 normalization value, “client demand” with 0.135 and “regulation and policy” with a normalization value of 0.297. All other challenges were considered critical, whereas “allocated funds to support” was the most critical challenge with a normalization value of 1.000.

With regard to the factors for applying I4.0 to GBs, regression analysis had to be conducted. However, this was preceded by the linearity test via SPSS 21.0. The linear relationship between dependent and independent variables is assumed to conduct a linear regression analysis. The results of the linearity test revealed a linear relationship between all independent variables and the dependent variable through the significance values of the deviation from linearity. The independent variable is considered linearly related to a dependent variable if it has significant values for the deviation from the mean that are greater than 0.05.

Since the linearity test was found to support the need for the regression analysis, the study determined *R* and *R* ^2^ values. *R*‐value (0.723) represents a simple correlation, indicating a high degree of correlation; *R* ^2^ = 0.523 shows how much the predictor variables technology, top management commitment, staff skills, infrastructure, financial arrangement, and operational control factors can account for the variation in the dependent variable (environmental performance). In fact, the regression shows associations, not proof that I4.0 causes improved environmental performance. It is suggested to perform longitudinal studies to track actual I4.0 adoption over time.

Lastly, the analysis of proposed strategies utilized mean, standard deviation, and PCA in SPSS 21.0. PCA evaluated communalities, which indicate the variance explained by underlying factors. Higher communalities suggest better representation by the factor model, whereas lower values point to unique or error variance. The lowest communalities were 0.641 for ST10 and 0.660 for ST4, but all strategies showed communalities above 0.50, confirming their acceptability. For instance, ST13 had a communality of 0.925, indicating 92.50% of its variance is explained by common factors. Consequently, with mean ratings between 4.19 and 4.48 and communalities from 0.591 to 0.875, all strategies are deemed reliable for implementing I4.0 in GBs.

### 5.2. Recommendations

GBs are essential because they can minimize the negative effects on the environment. Integrating green features into the design and construction of the building increases the efficient use of water, energy, and materials. Consequently, it is recommended that awareness of the potential of I4.0 application in GBs be emphasized to sustain such buildings. Despite the fact that GBs are certified by recognized bodies or agencies, including LEED certification by USGBC, there is an emphasis on implementing the proposed strategies. The proposed strategies include the emphasis on strengthening automation, IT infrastructures and technologies within the companies; setting up GB policies and plans that engage I4.0 technologies, and emphasis on investing in new technologies. Other relevant strategies include the provision of the relevant knowledge of the needed I4.0 hardware, emphasis on collaboration and partnership on digital technologies, sufficient funds to invest in digitalized equipment, having a clear vision of digital strategy, among other strategies. Regarding the factors for applying I4.0 technologies to GBs, all factors confirmed in this study should be adhered to. Such factors are mainly related to technology, top management commitment, employee skills, strengthening the available infrastructure, executing proper financial arrangements, and observing operational control factors. Likewise, the application of I4.0 should be guided by the proposed I4.0 strategies for GBs. The emphasis can begin with the four completed GB projects in Tanzania that are LEED‐certified by USGBC. Ultimately, it is expected that many processes involved in the construction of GBs through the use of I4.0 tend to lower costs while increasing productivity in the long run. I4.0 serves as the foundation for transforming how building projects operate and introducing real digital approaches in building construction.

### 5.3. Limitations and Future Research

Despite the assessment establishing awareness levels, factors for applying I4.0, and proposing strategies, the study focused only on the Dar es Salaam region. Consequently, generalizing the findings for other cities and countries may require additional variables based on the nature of the GBs. Only the completed buildings that were LEED‐certified by the USGBC were involved in the study, thus leaving the possibility of finding other GBs that might have been certified by other agencies or methods. For example, globally, there are some GBs which are certified via BREEAM (Building Research Establishment Environmental Assessment Method), LEED (Leadership in Energy and Environmental Design)–certified by USGBC, DGNB (a GB certification program created by the German Sustainable Building Council), CASBEE (Comprehensive Assessment System for Build Energy Environment Efficiency), among others. Also, the study only assessed the completed building projects; there is a gap in research on enabling GBs to be embodied with I4.0 technologies. Ultimately, a sustainability framework or guideline should assist GBs in adopting I4.0‐related technologies. Likewise, we acknowledge that the current conclusions may imply empirically validated performance improvements, whereas the study is primarily based on respondents′ awareness and projected outcomes rather than measured operational data. It is thus important to incorporate objective performance metrics—such as utility consumption records and waste management data—to validate these projections.

The study is also constrained by an unequal sample composition, with engineers and technicians predominating and architects, quantity surveyors, and clients being under‐represented. As a result, the findings are mostly based on the perceptions of technical specialists and may not fully represent the views of Tanzania′s larger GB stakeholder community. As a result, the findings should be regarded with caution and not applied outside the specific sample of respondents included in this study.

Lastly, for determining the factors for applying I4.0 technologies to GBs to enhance environmental performance, we acknowledge that, although variance inflation factor (VIF) values were within acceptable thresholds, the tolerance values indicate the possibility of moderate overlap among some independent variables (e.g., top management commitment and mission/vision). Additionally, we recognize that diagnostic tests for residual normality and heteroscedasticity, which are important in regression analysis, were not explicitly reported. In this study, the primary objective of the regression analysis was exploratory in nature, aimed at identifying key influencing factors rather than developing a predictive model. As such, although basic multicollinearity checks (VIF) were conducted to ensure no severe violations, more advanced diagnostics—such as correlation matrices, normality tests (e.g., Shapiro–Wilk), and heteroscedasticity tests (e.g., Breusch–Pagan), along with graphical assessments (Q–Q plots and residual scatterplots)—were not included in the current analysis. The absence of these additional diagnostic tests may affect the robustness of the regression assumptions and suggests that some degree of redundancy or unexplained variance among predictors cannot be fully ruled out. Future research will address this limitation by incorporating comprehensive diagnostic testing and model validation procedures to strengthen the reliability and generalizability of the findings.

## Author Contributions

Ismail W.R. Taifa: conceptualization, methodology, collected primary data, validation, visualization, writing—review and editing, project administration, resources, investigation, and formal analysis. Omari Mperella: conceptualization, investigation, funding acquisition, writing—original draft, methodology, validation, visualization, software, formal analysis, and data curation.

## Funding

No funding was received for this manuscript.

## Conflicts of Interest

The authors declare no conflicts of interest.

## Supporting information


**Supporting Information** Additional supporting information can be found online in the Supporting Information section. File S1: It provides some details of the closed‐ended questionnaire used to study to determine the awareness level of I4.0 technologies on GBs and identify factors influencing I4.0 application in GBs buildings.

## Data Availability

The data that support the findings of this study are available from the corresponding author upon reasonable request.
