# Supplementary material for: Enhancing Environmental Performance for Green Building Projects: Assessment Study due to the Applicability of Industry 4.0 Technologies
Source: ScientificWorldJournal. 2026 Jul 16;2026:4976856. doi: 10.1155/tswj/4976856 (PMC13373703; doi:10.1155/tswj/4976856)
Supplement: Supplementary file 1 — Supporting Information Additional supporting information can be found online in the Supporting Information section. File S1: It provides some details of the closed‐ended questionnaire used to study to determine the awareness level of I4.0 technologies on GBs and identify factors influencing I4.0 application in GBs buildings. [file TSWJ-2026-4976856-s001.docx]

**Questionnaire**

**The Awareness Level of Industry 4.0 on Green Buildings**

Please rate your Building using the following scale*:* 1 =Not at all familiar, 2=slightly familiar, 3 =moderately familiar, 4= Very familiar, 5=extremely familiar

| Please provide your ratings (1-5) for the following questions. | | 1 | 2 | 3 | 4 | 5 |
| --- | --- | --- | --- | --- | --- | --- |
| IG1 | Are you familiar with the term Industry 4.0? |  |  |  |  |  |
| IG2 | Are you familiar with the available Industry 4.0 technologies? |  |  |  |  |  |
| IG3 | Are you familiar with the term Green Buildings? |  |  |  |  |  |

Which of the following Industry 4.0 technologies are you aware of? Please rate your Building using the following scale*:* 1 =Very Unaware, 2 = Unaware, 3 = Neither Aware nor Unaware, 4 = Aware, 5=Very Aware

| **S/N** | **TECHNOLOGIES** | **1** | **2** | **3** | **4** | **5** |
| --- | --- | --- | --- | --- | --- | --- |
| AT1 | Artificial intelligence (AI) |  |  |  |  |  |
| AT2 | Cyber-Physical Systems |  |  |  |  |  |
| AT3 | Big data analytics |  |  |  |  |  |
| AT4 | Autonomous robotics |  |  |  |  |  |
| AT5 | Cyber security |  |  |  |  |  |
| AT6 | Building Information Modelling (BIM) |  |  |  |  |  |
| AT7 | Internet of things |  |  |  |  |  |
| AT8 | Cloud Computing |  |  |  |  |  |
| AT9 | Additive manufacturing (3D printing) |  |  |  |  |  |
| AT10 | Augmented reality |  |  |  |  |  |
| AT11 | Cyber manufacturing |  |  |  |  |  |
| AT12 | Virtual reality |  |  |  |  |  |
| AT13 | Blockchain technology |  |  |  |  |  |
| AT14 | Radio Frequency Identification (RFID) |  |  |  |  |  |

How would you rate the principles of green building you are familiar with? Please rate your building using the following scale: 1= Limited understanding, 2= Moderate understanding, 3=Good understanding, 4= Very good understanding, 5 = Expert understanding

|  | **Industry 4.0 technology** | **Level of agreement** | | | | |
| --- | --- | --- | --- | --- | --- | --- |
|  |  | **1** | **2** | **3** | **4** | **5** |
| PGB1 | Energy efficiency |  |  |  |  |  |
| PBG2 | Water conservation |  |  |  |  |  |
| PGB3 | Material selection |  |  |  |  |  |
| PGB4 | Indoor environmental quality |  |  |  |  |  |
| PGB5 | Waste reduction |  |  |  |  |  |

What is the industry 4.0 technologies that may be applied to green buildings in Tanzania? Please rate your Building using the following scale:1-Not Important, 2-Slightly Important, 3- Somehow Important, 4-Important, 5- Very Important

| **S/N** | **Technologies** | **1** | **2** | **3** | **4** | **5** |
| --- | --- | --- | --- | --- | --- | --- |
| IT1 | Artificial intelligence (AI) |  |  |  |  |  |
| IT2 | Cyber-Physical Systems |  |  |  |  |  |
| IT3 | Big data analytics |  |  |  |  |  |
| IT4 | Autonomous robotics |  |  |  |  |  |
| IT5 | Cyber security |  |  |  |  |  |
| IT6 | Building Information Modelling (BIM) |  |  |  |  |  |
| IT7 | Internet of things |  |  |  |  |  |
| IT8 | Cloud Computing |  |  |  |  |  |
| IT9 | Additive manufacturing (3D printing) |  |  |  |  |  |
| IT10 | Augmented reality |  |  |  |  |  |
| IT11 | Cyber manufacturing |  |  |  |  |  |
| IT12 | Virtual reality |  |  |  |  |  |
| IT13 | Blockchain technology |  |  |  |  |  |
| IT14 | Radio Frequency Identification (RFID) |  |  |  |  |  |

Rate the following challenges in implementing green building practices using the following scale: 1 = strongly disagree, 2 = disagree, 3 =neutral, 4 = agree and 5 strongly agree.

| **S/N** | **Challenges** | **Level of agreement** | | | | |
| --- | --- | --- | --- | --- | --- | --- |
|  |  | **1** | **2** | **3** | **4** | **5** |
| CGB1 | High initial cost |  |  |  |  |  |
| CBG2 | Awareness and Education |  |  |  |  |  |
| CBG3 | Infrastructure |  |  |  |  |  |
| CBG4 | Allocated funds to support |  |  |  |  |  |
| CBG5 | Lack of knowledge and skills |  |  |  |  |  |
| CBG6 | Availability of materials and technologies |  |  |  |  |  |
| CBG7 | Lack of Standardization and Certification |  |  |  |  |  |
| CBG8 | Culture and Aesthetics |  |  |  |  |  |
| CBG9 | Client demand |  |  |  |  |  |
| CBG10 | Regulation and policy |  |  |  |  |  |

Which Industry 4.0 technologies are employed for green building principles?

| S/N | **Technologies** | Green Building principles | | | | |
| --- | --- | --- | --- | --- | --- | --- |
|  |  | Energy  efficiency | Water  conservation | Waste  Reduction | Indoor Environmental  Quality | Material Selection |
| TG1 | Artificial intelligence |  |  |  |  |  |
| TG2 | Cyber-Physical Systems |  |  |  |  |  |
| TG3 | Big data analytics |  |  |  |  |  |
| TG4 | Autonomous robotics |  |  |  |  |  |
| TG5 | Cyber security |  |  |  |  |  |
| TG6 | Simulation |  |  |  |  |  |
| TG7 | Internet of things |  |  |  |  |  |
| TG8 | Cloud Computing |  |  |  |  |  |
| TG9 | Additive manufacturing (3D printing) |  |  |  |  |  |
| TG10 | Augmented reality |  |  |  |  |  |
| TG11 | Cyber manufacturing |  |  |  |  |  |
| TG12 | Virtual reality |  |  |  |  |  |
| TG13 | Blockchain technology |  |  |  |  |  |

**Factors for Applying Industry 4.0 to Green Buildings**

Based on your experience with green buildings, can you establish the factors for applying Industry 4.0 to Green Buildings? Scale: 1 = strongly disagree, 2 = disagree, 3 =neutral, 4 = agree and 5 strongly agree.

| **Component** | **Items** | **Factor/Statement** | **5-Likert Scale** | | | | |
| --- | --- | --- | --- | --- | --- | --- | --- |
|  |  |  | **1** | **2** | **3** | **4** | **5** |
| **Technology** | TC1 | Artificial intelligence (AI) |  |  |  |  |  |
|  | TC2 | Cyber-Physical Systems |  |  |  |  |  |
|  | TC3 | Big data analytics |  |  |  |  |  |
|  | TC4 | Autonomous robotics |  |  |  |  |  |
|  | TC5 | Cyber security |  |  |  |  |  |
|  | TC6 | Simulation |  |  |  |  |  |
|  | TC7 | The industrial internet of things |  |  |  |  |  |
|  | TC8 | Cloud Computing |  |  |  |  |  |
|  | TC9 | Additive manufacturing (3D printing) |  |  |  |  |  |
|  | TC10 | Augmented reality |  |  |  |  |  |
|  | TC11 | Cyber manufacturing |  |  |  |  |  |
|  | TC12 | Virtual reality |  |  |  |  |  |
|  | TC13 | Blockchain technology |  |  |  |  |  |
| **Top management commitment** | TMC1 | Does your firm's top management encourage the use of Industry 4.0? |  |  |  |  |  |
|  | TMC2 | Does the firm management understand how Industry 4.0 technologies are important for achieving sustainability in green buildings? |  |  |  |  |  |
|  | TMC3 | Does the firm's senior management and leadership offer industry 4.0 knowledge, support, and engagement? |  |  |  |  |  |
| **Mission/vision and strategy** | MV1 | The organization has a clearly defined mission and strategic plan that integrates Industry 4.0 technologies to achieve green building performance objectives. |  |  |  |  |  |
|  | MV2 | Is there a plan in place to arrange the steps necessary to put Industry 4.0 solutions into practice for Green Buildings? |  |  |  |  |  |
|  | MV3 | The organization has a documented roadmap with measurable targets for adopting Industry 4.0 technologies to improve energy efficiency, resource optimization, and overall green building project performance. |  |  |  |  |  |
| **Employee skills** | ES1 | Do the employees in your company know about Industry 4.0 technologies? |  |  |  |  |  |
|  | ES2 | Are the employees equipped to use Industry 4.0 technology? |  |  |  |  |  |
|  | ES3 | Are they competent enough to handle the industry 4.0 technologies? |  |  |  |  |  |
|  | ES4 | Do the employees at your firm recognize the benefits of implementing industry 4.0 technologies? |  |  |  |  |  |
| **Infrastructure** | IF1 | Do you concur that implementing Industry 4.0 requires the green building to have the necessary infrastructure? |  |  |  |  |  |
|  | IF2 | Are the ICT systems available to support data processing and decision-making? |  |  |  |  |  |
|  | IF3 | Is there reliable internet to support connectivity? |  |  |  |  |  |
| **Financial arrangement** | FA1 | The project allocates sufficient budget to acquire, implement, and maintain Industry 4.0 technologies (e.g., BIM, IoT, AI, digital twins) for green building projects. |  |  |  |  |  |
|  | FA2 | Adequate financial investment is provided for digital infrastructure, smart equipment, software, and environmentally sustainable technologies that support green building objectives. |  |  |  |  |  |
|  | FA3 | The project has access to financial incentives, grants, subsidies, tax benefits, or green financing mechanisms that encourage the adoption of Industry 4.0 technologies. |  |  |  |  |  |
|  | FA4 | Financial decisions regarding Industry 4.0 adoption are supported by systematic economic analyses, such as life-cycle costing, return on investment (ROI), and value-for-money assessments. |  |  |  |  |  |
| **Environmental factors** | EF1 | The extent to which Industry 4.0 technologies support compliance with environmental regulations, green building standards, and sustainability certifications through continuous monitoring and reporting. |  |  |  |  |  |
|  | EF2 | The ability of Industry 4.0 technologies (e.g., IoT, AI, BIM, and smart sensors) to optimize the use of energy, water, and construction materials while minimizing resource consumption and waste generation. |  |  |  |  |  |
|  | EF3 | The extent to which Industry 4.0 technologies enable monitoring and reduction of greenhouse gas emissions, construction waste, and environmental pollution throughout the building project lifecycle. |  |  |  |  |  |
